# The Effectiveness of Physical Activity-Promoting Web- and Mobile-Based Distance Weight Loss Interventions on Body Composition in Rehabilitation Settings: Systematic Review, Meta-analysis, and Meta-Regression Analysis

**DOI:** 10.2196/25906

**Published:** 2022-03-24

**Authors:** Heli Lahtio, Aki Rintala, Jaakko Immonen, Tuulikki Sjögren

**Affiliations:** 1 Faculty of Sport and Health Sciences The University of Jyväskylä Jyväskylä Finland; 2 Faculty of Social Services and Health Care LAB University of Applied Sciences Lahti Finland; 3 Department of Neurosciences Center for Contextual Psychiatry KU Leuven Leuven Belgium; 4 Faculty of Mathematics and Science The University of Jyväskylä Jyväskylä Finland

**Keywords:** technology, weight loss, rehabilitation, overweight, obesity, body mass index, waist circumference, body fat percentage, mobile phone

## Abstract

**Background:**

Overweight and obesity are major problems worldwide, and they lead to an increased risk for several diseases. The use of technology in the treatment of obesity is promising, but in the existing literature, there is considerable uncertainty regarding its efficacy. In this review, we included web- and mobile-based weight loss interventions that were implemented remotely in rehabilitation settings.

**Objective:**

The aim of this systematic review is to study the effectiveness of physical activity-promoting web- and mobile-based distance weight loss interventions in rehabilitation settings on body composition in comparison with control groups that did not use technology.

**Methods:**

Studies were searched from 9 databases. The inclusion criteria were as follows: population: age 18-65 years; intervention: physical activity-promoting web- and mobile-based distance weight loss interventions; comparison: control groups without the use of technology; outcome: changes in BMI, waist circumference, or body fat percentage; study design: randomized controlled trial. The quality of the studies was assessed by 2 researchers. Meta-analysis was performed, and we also conducted a meta-regression analysis to evaluate the factors associated with the changes in body composition outcomes if statistical heterogeneity was observed.

**Results:**

The meta-analysis included 30 studies. The mean quality of the studies was 7 of 13 (SD 1.9; range 3-10). A statistically significant difference was observed in BMI (mean difference [MD] 0.83, 95% CI 0.51-1.15 kg/m^2^; *P*<.001), waist circumference (MD 2.45, 95% CI 1.83-3.07 cm; *P*<.001), and body fat percentage (MD 1.07%, 95% CI 0.74%-1.41%; *P*<.001) in favor of the weight loss groups using web- or mobile-based interventions. Meta-regression analyses found an association between personal feedback and BMI (*P*=.04), but other factors did not play a role in explaining statistical heterogeneity.

**Conclusions:**

Web- and mobile-based distance weight loss interventions significantly reduced BMI, waist circumference, and body fat percentage. Future studies should focus on the comparability of the intervention content. Future studies are needed to better understand weight loss and identify which components are essential in achieving it.

**Trial Registration:**

PROSPERO CRD42016035831; https://tinyurl.com/7c93tvd4

## Introduction

### Background

Overweight and obesity are conditions that may influence people’s health [1]. In 2016, more than 1.9 billion adults were overweight and 650 million were obese [1]. Overweight is defined as a BMI of ≥25 kg/m^2^, and obesity is defined as a BMI of ≥30 kg/m^2^ [1]. However, the BMI categories seem to differ based on cultural and racial backgrounds. For example, in Asian populations, the BMI categories are as follows: <18.5 kg/m^2^ is underweight, 18.5 kg/m^2^ to 23 kg/m^2^ is increasing but acceptable risk, 23 kg/m^2^ to 27.5 kg/m^2^ is increased risk, and ≥27.5 kg/m^2^ is high risk [2]. BMI is widely used to define overweight and obesity because it is simple to use, and it is the same for adults of all genders and ages [1]. However, BMI does not consider the distribution of fat, and it does not separate the weight of muscle mass from that of fat mass [3].

Waist circumference has been used to identify abdominal fat distribution [3]. Abdominal fat is metabolically more active than fat in the hips or thighs [3]. However, waist circumference is an imprecise indicator because it does not separate subcutaneous fat from visceral fat [4]. Another limitation is that the cutoff points cannot be applied universally [5]. However, increased waist circumference has been shown to be associated with cardiovascular diseases such as hypertension [5]. Body fat percentage provides valuable information about the distribution of fat [3]. Bioimpedance, dual-energy x-ray absorptiometry (DXA), and skinfold thickness are generally used, but their reliability has been called into question. For example, Sun et al [6] found that bioimpedance was reliable in people with normal body fat range, but it tended to underestimate body fat in people who were thin, overweight, or obese. Bioimpedance measures the electrical properties of body tissues as alternating electric current flows through a human body using parameters such as fat-free mass or total body water [7]. Another example is DXA, which is a 3-component model (1 component of fat mass and 2 components of fat-free mass), but it is not widely used because of its high cost and risk of unnecessary radiation exposure for patients [8].

Several studies have highlighted the role of the combination of physical activity and nutrition in weight loss [9-12]. However, physical activity and nutrition are not the only features affecting weight loss. For example, the gastrointestinal tract, genetics, psychological stress, and medication also affect weight loss [13]. Physical activity has a large role in weight loss in maintaining the amount of fat-free mass and improving the body composition of participants [14]. In addition, it has many health benefits [10,15,16], such as improving the cardiorespiratory system and muscular strength, that are associated with health and functional capacity [17]. It also improves blood pressure and increases insulin sensitivity and fat oxidation [15]. Physical inactivity increases the risk of several diseases [16,18] as does overweight [3].

Physical activity is an important factor in the prevention and treatment of noncommunicable diseases such as stroke, heart diseases, diabetes, and breast and colon cancer. It is also important in the prevention of risk factors of noncommunicable diseases, such as hypertension. Physical activity can be defined as a movement that has been produced by the skeletal muscles and requires energy expenditure [19]. Physical activity is an integral part of rehabilitation because it has a variety of effects on many organs and functions, such as lowering blood pressure [20]. Physical activity-based rehabilitation has been used, for example, in home-based rehabilitation [20], cardiac rehabilitation [21], and spinal cord injury rehabilitation [22], as well as with various chronic disabilities (eg, musculoskeletal or neurological problems) [23]. Rehabilitation plays a critical role in preventing as well as in minimizing the limitations of functioning that are associated with different conditions [24]. Rehabilitation is needed by anyone with health conditions, not only people with disabilities [24]. In this review, we were interested in weight loss interventions in rehabilitation settings and because of the role of physical activity in rehabilitation [19,25], we included only studies that included physical activity.

The use of technology has increased in the rehabilitation environment in the past decade. For example, technology has shown benefits for rehabilitation focused on cardiac rehabilitation [26,27], nonalcoholic fatty liver disease [28], serious mental illness [29,30], and older adults [31]. These studies focused on weight loss [27-29,31], lifestyle changes [30], and decreasing coronary risk factors [26]. The studies contained, for example, mobile health technology (eg, smartphones and SMS text messages) and social media to increase motivation and facilitate self-monitoring and peer support [29]; a Facebook group-based lifestyle program where participants were able to connect and support each other with regard to healthy eating and exercise goals [30]; an SMS text messaging intervention where the messages provided education on nutrition, exercise, and stress management, as well as improving motivation [28]; videoconferencing sessions consisting of nutrition and exercise sessions [31]; and mailed written materials, a pedometer, and coaching and goal-setting sessions through the telephone [27]. The study by Varnfield et al [26] included smartphones for monitoring health and exercise and delivered motivational and educational materials. It also provided access to a web portal where participants received weekly consultations [26].

Many systematic reviews have studied the effectiveness of technology-based distance weight loss interventions among adults [32-38], but only in a few reviews was the primary outcome related to body composition [32-34]. In other reviews, the primary outcome was weight change [35-38]. There is evidence that technology-based distance weight loss interventions improved weight loss and body composition outcomes compared with usual care without the use of technology [35-37], no treatment [34] or minimal intervention (information) [33], pamphlets [34], and self-help written materials (eg, dietary guidelines) [35]. There was a variety in the selected study designs in the previous systematic reviews. For instance, most reviews included randomized controlled trials (RCTs) [32-38], but quasi-RCTs [34], single-group intervention studies [36], non-RCTs [36], comparative effectiveness trials [36], retrospective cohort studies [36], and pre-post and quasi-experimental studies [32] were also included. There was variation in the inclusion criteria of the reviews in terms of control groups. Two reviews [34,35] determined what content they would accept for the control groups. They included usual care [34,35], educational materials [34], telephone interventions [34], in-person interventions [34,35], a no-intervention control group [35], or another eHealth intervention [35]. One review [32] had no restrictions about the content of the control group, 2 reviews [33,37] did not mention the content of the control group in the inclusion criteria, and in 2 reviews [36,38], all the studies did not include a control group. There was statistical heterogeneity in most of the reviews [32-37].

Only three previous reviews have used body composition outcomes: BMI [32,34] and waist circumference [33,34]. None of these reviews included all body composition outcomes (BMI, waist circumference, and body fat percentage). Other reviews investigated solely the effectiveness of weight loss (measured in kilograms) [35-37]. Although these systematic reviews have presented positive results, there is also contrary evidence. In body composition outcomes, no difference between the technology user group and control group was found [32]; technology-based interventions achieved smaller weight loss than in-person interventions [34]; or there were inconsistent results [38]. Previous reviews have been heterogeneous in terms of technology. They have described the technology used as eHealth interventions [32,35,36], internet-based interventions [33,37,38], an intervention that used a PC or mobile device [37], or an interaction-enabled computer-based intervention [34]. This review has focused on web- and mobile-based interventions, and the outcome variables were BMI, waist circumference, and body fat percentage.

Only 1 previous systematic review [33] has used meta-regression analysis to investigate the association between personal characteristics and waist circumference. The authors found that baseline waist circumference, gender, and social support were significantly associated with a reduction in waist circumference [33]. However, the meta-regression analysis only focused on waist circumference and studies that included internet-based interventions. Similar meta-regression analyses have not been conducted on other outcomes such as BMI and body fat percentage, which may increase the knowledge that should be considered in future treatment strategies for weight loss interventions. This meta-regression analysis provides a more comprehensive analysis than other previous reviews. In addition, this review discusses how different studies have implemented distance weight loss interventions that use web- and mobile-based intervention.

It has been suggested that technology-based distance health promotion interventions should be theory-based [39] and strengthen self-regulatory skills, which are essential in maintaining behavior change [40]. Widely used theories in technology-based weight loss interventions are cognitive behavioral [41] and social cognitive theories [40]. The cognitive behavioral theory is based on the assumption that all behavior is learned and internal and environmental factors are related to the behavior. It teaches to explore, identify, and analyze dysfunctional patterns of thinking and acting. Strategies related to cognitive behavioral theory include self-monitoring, goal setting, and social support. [41]. According to the social cognitive theory, people learn by observing social interactions and experiences. According to the social cognitive theory, effective strategies are demonstration, modeling, and social support [41].

### Objective

The aim of this systematic review is to study the effectiveness of physical activity–promoting web- and mobile-based distance weight loss interventions in rehabilitation settings on body composition in comparison with control groups that do not use technology. We conducted a comprehensive systematic review with meta-analysis of absolute changes in BMI, waist circumference, and body fat percentage. We also conducted a meta-regression analysis to evaluate the factors associated with the changes in body composition outcomes if statistical heterogeneity was observed.

## Methods

### Data Sources

The following 9 databases were searched from January 2000 to January 2016: PsycINFO, Cochrane Central Register of Controlled Trials, Embase, CINAHL, Ovid MEDLINE, OTseeker, PEDro, Web of Science, and PubMed. An updated search of the following 5 databases was performed from January 1, 2016, to March 31, 2020: PsycINFO, CINAHL, Ovid MEDLINE, PEDro, and PubMed. It was decided to conduct the updated search only in the databases from which we retrieved all the included studies in the first search. Our updated search resulted in 2684 studies, and this number is considered sufficient; it is unlikely that the updated search would have resulted in missing out on studies relevant to our review. In all, 2 information specialists (HL and AR) performed the searches using search terms related to technology and physical activity as well as terms reflecting RCT and clinical trial study designs. The search strategy is presented in Multimedia Appendix 1. A manual search was conducted using the reference lists acquired from the studies found in the search. This systematic review is registered with PROSPERO (CRD42016035831).

### Study Selection

With the support of a research team, 1 researcher (HL) was responsible for searching for studies related to the outcomes of this study. The studies were screened by 1 reviewer (HL) using the Population, Intervention, Comparison, Outcomes, and Study Design (PICOS) approach recommended in the PRISMA (Preferred Reporting Items for Systematic Reviews and Meta-Analyses) checklist [42]. The inclusion criteria were set according to the PICOS framework and were as follows: population: adults aged 18˗65 years; intervention: physical activity–promoting web- and mobile-based distance weight loss interventions in rehabilitation settings in the experimental group; comparison: control group without the use of technology; outcome: changes in BMI, waist circumference, or body fat percentage; and study design: RCT. We decided to set the upper age limit as 65 years because Finnish legislation categorizes people aged >65 years as elderly [43]. Rehabilitation is defined as a set of interventions designed to optimize functioning and reduce the disability of individuals who have health conditions [14]. As this review explores the benefits of web- and mobile-based interventions in rehabilitation settings, the interventions in the included studies had to involve participants requiring rehabilitation. We defined web-based interventions as interventions that are performed through the internet and mobile-based interventions as interventions that are performed through mobile devices [44]. Interventions had to be implemented remotely so that the participant was able to use the technological device without the presence of the health care provider. The only exceptions were monthly measurement visits or introduction lessons, seminars, or discussion visits. As outcome variables, we used European BMI categories. However, in different racial groups, BMI categories differ. Studies published in Finnish, English, or Swedish were included. In the reporting of the review, the PRISMA guidelines [42] were adhered to.

### Methodological Quality of the Included Studies

The methodological quality of the RCTs was evaluated using the 13-point scale introduced in the guidelines for systematic reviews in the Cochrane Collaboration Back Review Group [45]. Two assessors (HL and AR) from the research group evaluated the studies. Both assessors evaluated the studies independently at first, after which the results were discussed. If necessary, a third reviewer (TS) was consulted to resolve any disagreement. However, a consensus was reached for each study. If a criterion was fulfilled, the domain was considered a *Yes* and counted as a score. If a criterion was not fulfilled, it was considered a *No*. If a criterion was unclear, it was considered a *Don’t know*, symbolized by *?.* The main quality domains were the following: A1: randomization; B2: concealed treatment allocation; C3: blinding of the patients; C4: blinding of the care providers; C5: blinding of the outcome assessors; D6: dropout rate; D7: analysis of participants in the groups to which they have been assigned; E8: selective outcome reporting; F9: similarity of groups at baseline; F10: similarity or absence of cointerventions; F11: compliance; F12: timing of outcome assessments, and F13: other types of biases. After summing the *Yes* scores, the maximum score obtainable was 13. The quality points were used as a covariate in the meta-regression analysis.

### Data Analysis

The meta-analysis consisted of mean difference (MD) analyses for three parameters: BMI, waist circumference, and body fat percentage. A positive MD was considered to favor the experimental group. MD was calculated using groupwise MDs; if these groupwise MDs (and corresponding SDs or SEs) were reported inadequately, only end point measures were used. Instead of SDs or SEs, some studies reported *P* values or CIs. In this case, the corresponding SDs were calculated from *P* values or CIs, assuming a 2-tailed *t* test. If the median was reported instead of the mean, the median was used to estimate the mean by using the formula range/4 [46]. The authors of the included studies were contacted if the required data were missing or not adequately reported. Responses were received from 11 authors.

All 3 analyses were conducted in 2 phases. First, an ordinary meta-analysis (fitted with restricted maximum likelihood) with no covariates was conducted to estimate the average MD, the heterogeneity among the studies, and the possibility of publication bias. Analyses were conducted using R with a meta-analysis package (metafor; The R Foundation for Statistical Computing) [47]. The level of heterogeneity was measured using the I^2^ measure, and its significance was tested using the Cochran Q test. Thresholds for the interpretation of the I^2^ measure were as follows: low (0%-40%), moderate (30%-60%), substantial (50%-90%), and considerable (75%-100%) [48]. Publication bias was assessed visually using funnel plots and by using a regression test. The regression test measures the association between the effect sizes and the corresponding SEs [49]. If the funnel plot is symmetrical, publication bias does not exist [48].

Second, the influence of covariates was studied using meta-regression analysis. Forward selection starting with a model without covariates was used. Model fit was measured by corrected Akaike information criterion (AICc): the smaller the AICc, the better the model fit. Models were fitted with maximum likelihood, which enables model comparison. Finally, the model to be chosen (if not the same as in the first phase) was fitted with restricted maximum likelihood to produce unbiased *P* values and CIs. A 3-level meta-analytic model [50] was applied in both phases. Dependence induced by multiarm studies was treated with the method proposed in the study by Gleser and Olkin [51].

Meta-regression analysis was used to explain the statistical heterogeneity. A prior decision was made to investigate the study-level characteristics. Covariates were related to the PICOS framework: population: mean age of the participants, the proportion of men, and analysis of the prevention; intervention: length of the intervention, personal goals, self-reporting, personal feedback, and using theory; comparison: the content of the control group; study design: quality of the studies (for the coding of these variables, see Table 1).

The 9 covariates chosen for the meta-regression analysis were based on the findings from previous systematic reviews. Covariates related to the population were based on mixed findings of previous studies. The results were mixed with regard to weight loss and age [52,53] or gender [37,52,54] of the participants. In previous reviews, too, the results were mixed in studies concerning primary prevention [34], secondary prevention [33,55,56], and tertiary prevention [52,57]. Primary prevention means that a participant is healthy and the prevention is targeted at preventing diseases. In secondary prevention, a participant has symptoms of a disease, and the actions are targeted to prevent more symptoms or the development of a disease. For example, a participant may have increased blood pressure and the actions are targeted to prevent hypertension. Tertiary prevention means that a participant already has a disease such as hypertension and the actions are targeted to decrease the effect of the disease [58]. Regarding the interventions, the duration of weight loss interventions [52,59] and the best methods to achieve greater weight loss [34,57,60] were considered. Previous systematic reviews have indicated that self-reporting [34,61], intervention personalization [61], in-person feedback, and targeted structured lifestyle coaching are important in facilitating weight loss [34]. Because of these results, personal goals, self-reporting, and personal feedback were chosen as covariates. Previous reviews [33,37,38] have observed that several studies use theories; therefore, using a theory was also chosen as a covariate. Regarding comparison, we decided to study whether the content of control groups influences the heterogeneity. In this review, the content of the control groups varied greatly. A study by Johns et al [62] found that control groups who received more advice or counseling lost more weight than control groups who received, for example, only pamphlets. We generated five categories that were compared with a wait-list or no intervention (0): (1) usual care, (2) usual care plus minimal guidance (eg, pamphlets), (3) paper instructions, (4) paper instructions plus minimal instructions (eg, introduction lesson); and (5) other intervention concerning weight loss. Finally, we included a covariate reflecting the level of study quality because previous systematic reviews have discussed the variability of the quality of the studies [33,38,57,60,63] and the heterogeneity of the studies [33,55-57].

**Table 1:** Description and coding of the covariates used in the meta-regression analysis.

Covariate	Description	Coding
Mean age	Mean age of the participants	Years (continuous variable; range 20.0-69.1)
Proportion of men	Relative proportion of men from 0% to 100%	Range 0-1; the scale has been changed
Analysis of prevention	Secondary and tertiary prevention were compared with primary prevention	S: secondary prevention; T: tertiary prevention
Length of the intervention	Length of the intervention	Weeks (continuous variable; range 4-8)
Personal goals	If the intervention included setting personal goals for the weight loss intervention	No (0) or Yes (1; dichotomous variable)
Self-reporting	If the intervention included self-reporting of values needed in the study (eg, weight, steps, and diet)	No (0) or Yes (1; dichotomous variable)
Personal feedback	If the participants received personal feedback about their progress (eg, weight loss or physical activity)	No (0) or Yes (1; dichotomous variable)
Using theory	If the intervention used motivational or behavior change theories	No (0) or Yes (1; dichotomous variable)
Content of the control group	0=wait-list or no-intervention group 1=usual care or content of the control group has not been mentioned 2=usual care plus minimal guidance (eg, pamphlets) 3=paper instructions 4=paper instructions plus minimal instructions (eg, introduction lessons) 5=other intervention concerning weight loss (eg, annual physician appointment and fitness test)	Six-level factor using the wait-list group or no-intervention group as a reference group (categorical variable)
Quality of the studies	The level of quality assessment	Range 0-13 (continuous variable) [38]

## Results

### Overview

**Figure 1:**
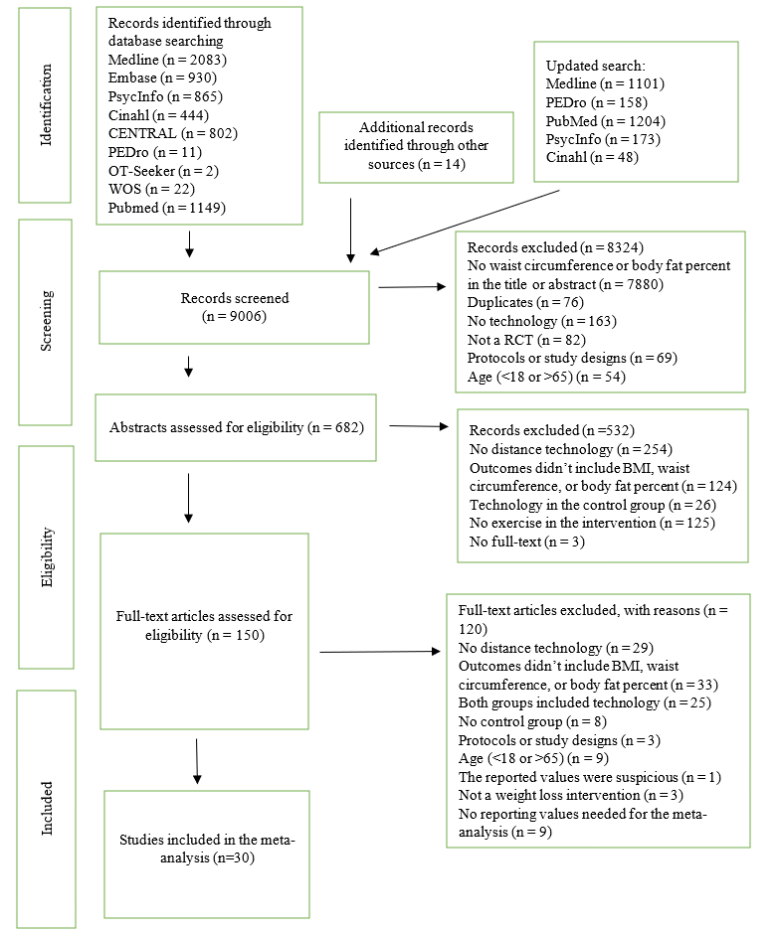
Flowchart of the study selection.

The search strategy yielded a total of 9006 potentially relevant studies, of which 8976 (99.67%) were excluded, meaning 30 (0.33%) studies met the inclusion criteria and were included in the meta-analysis and meta-regression analysis. A detailed description of the citation screening and selection process is presented in Figure 1.

Among the 30 included studies, the outcome variable was BMI in 19 (63%) studies [64-82], waist circumference in 25 (83%) studies [64,66,68-76,78-91], and body fat percentage in 11 (37%) studies [64,70,72,73,76,78,80,86,89,92,93]. All outcome variables were included in 20% (6/30) of the studies [64,72,74,76,78,80]. In the 11 studies using body fat percentage as an outcome, 7 (64%) used bioimpedance [64,72,73,76,78,89,93], 1 (9%) used DXA [86], and 1 (9%) used skinfold [80], whereas in 2 (18%) studies, the methods were not mentioned [70,92]*.* A detailed description of the included studies is presented in Multimedia Appendix 2 [43-72].

### Methodological Quality of the Studies

The mean methodological quality of the studies was 7 out of 13 (SD 1.9; range 3-10). The quality assessment revealed great variability in the quality of the studies, with the most frequent source of bias being insufficiently reported blinding of the participants or care provider. There was also insufficient reporting on compliance with the intervention, avoidance of cointerventions, dropout descriptions, and analysis of participants in the assigned groups. The methodological quality of the studies is presented in Multimedia Appendix 3 [64-93].

### Description of the Participants

A total of 6103 participants were included in the studies, and the mean percentage of men was 42% (SD 7.7%). The mean age of the participants was 40.2 (SD 17.1) years. In a study by Sakane et al [79], the average age was not reported, but the age range of the participants was used. Therefore, the average age was imputed from the average age of Japanese people aged 20-65 years [94], weighted with the proportions of men and women in the study. A similar imputation was made by Anderson et al [65], except only the lower bound (18 years) was reported; the average age of American people aged 18-84 years [95], weighted with the proportions of men and women, was used. The number of participants in the experimental groups was 3624 (mean 88.4, SD 98.4), and the mean percentage of men was 38.8% (SD 31.2%). The number of participants in the control groups was 2490 (mean 80.3, SD 106.8), and the mean percentage of men was 41.1% (SD 31.8%). A detailed description of the participants is presented in Multimedia Appendix 2.

### Description of the Interventions

The mean duration of the interventions was 30.4 weeks (SD 17.9; range 4 weeks to 2 years). In the included studies, weight loss was set as the main aim of the study in 60% (18/30) of the studies [64,66,68,71-74,76-82,85,86,89,91]. Of the 30 included studies, 3 (10%) aimed to increase the level of physical activity [70,88,92], whereas 2 (7%) focused on both increasing the participants’ physical activity and decreasing their weight [84,93]. Of the 30 included studies, 6 (20%) aimed at more specific interventions such as disease management in diabetes [65], lifestyle modification [67], reducing blood pressure [69], health management [83], reducing cardiovascular risk and weight loss [90], and improving quality of life [87]. The included studies were implemented in rehabilitation settings. The instructions for the intervention were given by a health care professional (eg, general practitioner or physician), or the studies were performed in the field of health science (eg, a department of health and physical activity). A primary outcome was weight loss in 37% (11/30) of the studies [64,66,67,71,72,74,76,79,80,82,86], both weight loss and physical activity in 33% (10/30) of the studies [68,73,77,78,81,84,89-91,93], and physical activity in 10% (3/30) of the studies [70,88,92]. Weight change was a secondary outcome in 7% (2/30) of the studies [65,87], and weight loss without definition was the primary or secondary outcome in 10% (3/30) of the studies [69,83,85]. A detailed description of the interventions is presented in Multimedia Appendix 2.

The included studies used mainly mobile- or web-based interventions. Of the 30 included studies, 5 (17%) [65,71,85,87,90] used only mobile phones and 3 (10%) [70,79,83] were only web-based. Both mobile- and web-based interventions were included in 30% (9/30) of the studies [66,69,72,74,75,77,78,80,82]. Altogether, a mobile-based intervention was included in 73% (22/30) of the studies [65,66,68,69,71-75,77,78,80,82,84-91,93], whereas a web-based intervention was included in 60% (18/30) of the studies [64,66,68-70,72,74-80,82,83,87,91,92]. Most of the studies used combinations of technological devices, such as email in 17% (5/30) of the studies [66,68,75,82,91], pedometers in 37% (11/30) of the studies [64,69,73,76,77,84,86,88,89,91,93], and DVDs in 10% (3/30) of the studies [64,69,76]. Other technologies used were a step counter [66], an activity tracker [67], an activity-measuring device [78], digital scales [84], video clips [74], and an armband [80]. Of the 30 studies, 4 (13%) combined a web-based intervention with a step counter [66] or pedometers [64,76,91], 9 (30%) combined a mobile-based intervention with an activity tracker [67] or pedometers [69,73,84,86,88,89,91,93], and 2 (7%) used both web- and mobile-based interventions with a pedometer [77] or an activity-measuring device [78].

There was a large variation in the frequency of technology use. The participants were contacted by telephone every week [77], every other week [64,71,88,90], every 4-6 weeks [87], or 4 and 8 weeks after baseline measurements [72]. In the study by Rimmer et al [77], telephone calls were reduced from weekly calls to monthly calls, and in the study by Anderson et al [65], call frequency depended on the risk stratification of the participants. There was also variation in the guidance regarding the frequency of using websites, smartphone apps, emails, and accelerometers as a technology. In the studies using websites and smartphone apps, guidance for logging in varied from daily use [66,69,81], use at least once a week [74], use 3-4 times per week [76,92], and use at least five times per week [72]. Of the 30 studies, 5 (17%) did not report the frequency of using a website [68,78,80,83,91]. The frequency of emails was weekly in 7% (2/30) of the studies [68,76]. In the studies using pedometers and accelerometers, guidance for wearing them varied from daily use [69,73,84,86,88] to 2 days a week [64,76,80,89,92]. In the study by Rimmer et al [77], the frequency was not reported.

There was variation in the content of the control groups. The control group included usual care in 27% (8/30) of the studies [65,74,82,84,88,90,92,93], a wait-list in 20% (6/30) of the studies [66,68,76,77,83,85], and minimal treatments such as brochures [67,69,71,86,87,90,91] or an annual physician’s examination [72,87] in other studies. Group meetings [78,86], introductory sessions [69], and mailed feedback about the baseline assessment [89] were also used as content in the control groups. Of the 30 studies, 9 (30%) [66,68,69,76,78-80,86,91] included >1 intervention group, all of which were compared with the same control group.

### The Effectiveness of Web- and Mobile-Based Distance Weight Loss Interventions on BMI Compared With Control Groups Without the Use of Technology

**Figure 2:**
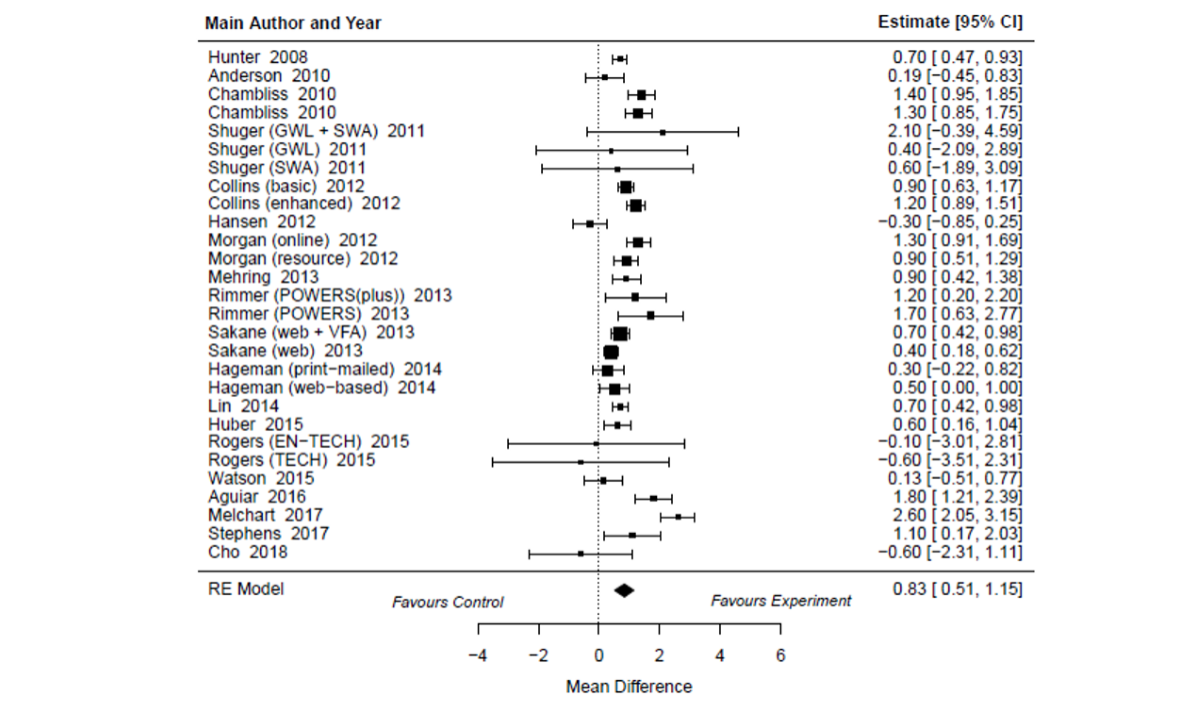
Forest plots describing the effectiveness of web- and mobile-based weight loss intervention on BMI compared with control groups without the use of technology [64,70,72,73,76,78,80,86,89,92,93]. EN-TECH: enhanced technology-based system; GWL: group-based behavioral weight loss program; GWL + SWA: group-based behavioral weight loss program + The SenseWear Armband; RE: random effect; SWA: The SenseWear Armband; TECH: technology-based system.

Web- and mobile-based distance weight loss interventions were 0.83 units more beneficial to BMI than the control groups without the use of technology (MD 0.83, 95% CI 0.51-1.15 kg/m^2^; *P*<.001; Figure 2). The studies were considerably heterogeneous (I^2^=90%; *P*<.001). On the basis of the regression test, there were no signs of publication bias (*P*=.61). According to the AICc, studies using personal feedback have an impact on BMI reduction compared with the included studies that did not have personal feedback (0.32, 95% CI 0.02-0.62; *P*=.04). However, other covariates did not have any effect on the reduction in BMI. Detailed information about the covariates of BMI is presented in Multimedia Appendix 4, and the analysis of BMI is presented in Multimedia Appendix 5 [43-61].

### The Effectiveness of Web- and Mobile-Based Distance Weight Loss Interventions on Waist Circumference Compared With Control Groups Without the Use of Technology

**Figure 3:**
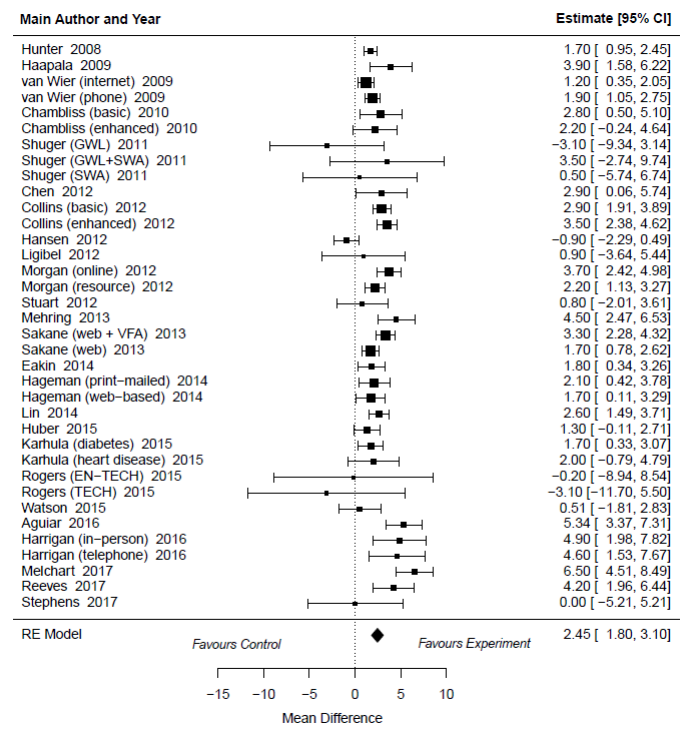
Forest plots describing the effectiveness of web- and mobile-based weight loss intervention on waist circumference compared with control groups without the use of technology [64,66,68-76,78-91]. EN-TECH: enhanced technology-based system; GWL: group-based behavioral weight loss program; GWL + SWA: group-based behavioral weight loss program + The SenseWear Armband; RE: random effect; SWA The SenseWear Armband; TECH: technology-based system; VFA: visceral fat measurement group.

Web- and mobile-based distance weight loss interventions were 2.45 units more beneficial to waist circumference than the control groups without the use of technology (MD 2.45, 95% CI 1.83-3.07 cm; *P*<.001; Figure 3). Substantial heterogeneity was observed (I^2^=78%; *P*<.001). On the basis of the regression test, there were no signs of publication bias (*P*=.73). According to the AICc, none of the covariates explained the variability in waist circumference reduction (Multimedia Appendices 4 and 6 [43,45,47-55,57-70]).

### The Effectiveness of Web- and Mobile-Based Distance Weight Loss Interventions on Body Fat Percentage Compared With Control Groups Without the Use of Technology

**Figure 4:**
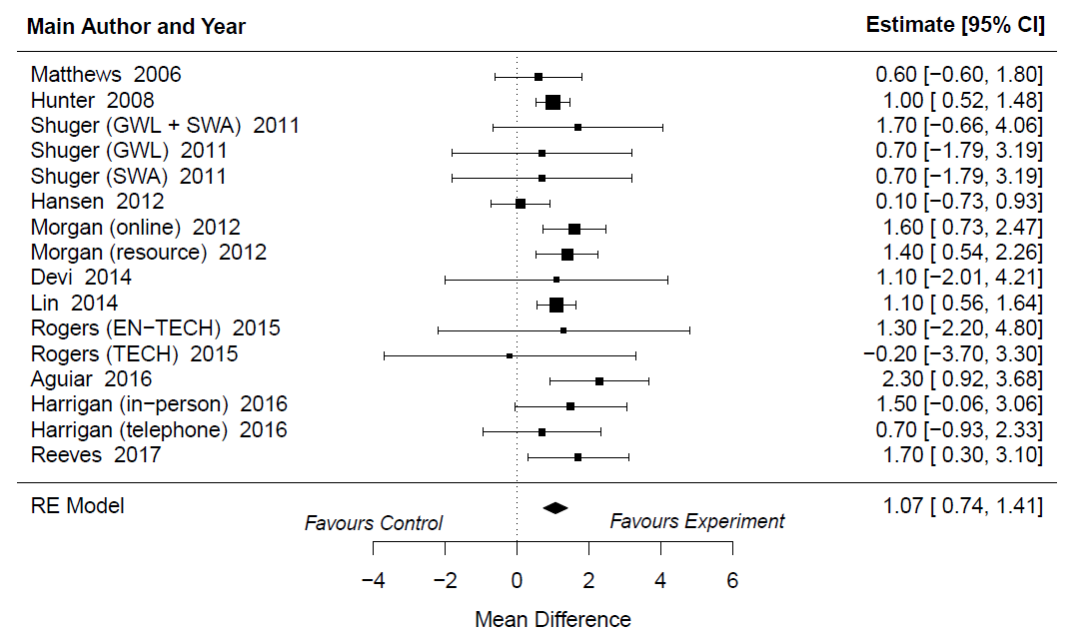
Forest plots describing the effectiveness of web- and mobile-based weight loss intervention on body fat percentage compared with control groups without the use of technology [64,70,72,73,76,78,80,86,89,92,93]. EN-TECH: enhanced technology-based system; GWL: group-based behavioral weight loss program; GWL + SWA: group-based behavioral weight loss program + The SenseWear Armband; RE: random effect; SWA: The SenseWear Armband; TECH: technology-based system.

Web- and mobile-based distance weight loss interventions were 1.07 units more beneficial to body fat percentage than the control groups without the use of technology (MD 1.07%, 95% CI 0.74%-1.41%; *P*<.001; Figure 4). The heterogeneity was low (I^2^=18%; *P*=.54). On the basis of the regression test, there were no signs of publication bias (*P*=.81). According to the AICc, none of the covariates explained the variability in body fat percentage. However, the quality of the studies indicated that it might have an impact on the reduction in body fat percentage, but it was not statistically significant (estimate 0.12, 95% CI –0.01 to 0.25; *P*=.06; Multimedia Appendices 4 and 7 [43,49,51,52,55,57,59,65,68,71,72]).

## Discussion

### Principal Findings

This systematic review and meta-analysis indicated a statistically significant difference in BMI, waist circumference, and body fat percentage in favor of the physical activity-promoting web- and mobile-based distance weight loss experimental groups when compared with the control groups without the use of technology. Previous systematic reviews have also observed that weight loss [32,33,38] and waist circumference reduction [38] were significantly greater in technology-based experimental groups when they were compared with usual care without the use of technology or with minimal interventions (eg, pamphlets). Although in our meta-analysis, the experimental groups achieved greater reduction in BMI, waist circumference, and body fat percentage, the reductions in all outcome variables were relatively small and not clinically meaningful. Previous systematic reviews have indicated similar results [32,96]. They found that although the results were statistically significant, they were not clinically meaningful.

Despite using a vast number of different covariates in our meta-regression analysis, only personal feedback showed a trend toward an effect on the variability of the body composition outcome measurements. Our meta-regression analysis did not indicate that the level of study quality affected the findings, although it was close to statistical significance. This is reassuring for researchers who are conducting RCTs to investigate weight loss. Variability was also considered large in all meta-regression analyses; therefore, a firm scientific conclusion is challenging to draw. The review by Seo et al [33] found that mean waist circumference at baseline, proportion of male participants, and social support were related to significant waist circumference changes. Other intervention content-related covariates were not significant in the reduction in waist circumference. In the review, the mean age of the participants, existing diseases, and the status of general obesity also had a significant effect on waist circumference reduction [33]. Despite important elements found in previous studies (self-monitoring [8,61], in-person feedback [34], targeted and structured lifestyle coaching [34,61], program use [61], and social support [61]), only personal feedback and the quality of the studies were related to greater changes in body composition in our findings. Khaylis et al [97] qualitatively studied components that are effective in facilitating weight loss. According to the researchers, self-monitoring, counselor feedback and communication, social support, structured programs, and individually tailored programs are effective in technology-based weight loss interventions [97]. In this systematic review, self-reporting, personal goals, and personal feedback were used as covariates, but the study did not find an effect on body composition.

In this review, the interventions of the experimental groups were web- or mobile-based. However, only 27% (8/30) of the studies [65,70,71,79,83,85,87,90] used solely web- or mobile-based technology, whereas the other studies used multiple technologies in their interventions combining web- or mobile-based technology with, for example, a pedometer [88], or a DVD and pedometer [76]. In the included studies, there were none in which the experimental group and control group had similar interventions, with the only difference being that the experimental group used technology and the control group did not. In the experimental group, the content of the intervention might have included several methods, such as telephone calls, self-reporting, personal feedback, and pedometers, whereas the control group had only brochures. Therefore, it is difficult to identify the real effects of technology-based distance weight loss interventions on body composition. In addition, several other factors, such as those related to motivation and commitment to the intervention, could have affected the results. However, in rehabilitation, communication between the participant and health care provider is essential. Although we recognized that the level of communication and social support may complicate investigating the effectiveness of the technology itself, in rehabilitation settings this is unavoidable. Therefore, we included motivation with personal feedback and personal goals as a factor in the meta-regression analysis because this has been found to be a contributing factor in weight loss interventions [97-99]. According to our findings, these covariates did not influence the results of this study and only personal feedback had a statistically significant result in decreasing BMI.

This review provides insight into how weight loss interventions can be implemented in rehabilitation settings, both with and without the use of technology. We explored the benefits of the web- and mobile-based weight loss interventions that were mainly implemented remotely and promoted physical activity. The interventions included, at minimum, access to a basic web-based program [68] or telephone calls [65]. The most intensive intervention included, for example, a meeting with the health educator, individual reports of measurements, a 2-hour group seminar, basic guidance for healthy eating and physical activity, self-monitoring of daily food intake and physical activity, weekly individual feedback reports on diaries, step counters, monthly email newsletters, telephone consultations, and monthly clinic visits with discussions about behavioral strategies [66]. There was also variation in the content of the control groups. Usual care and wait-list were most commonly used. However, pamphlets, annual physician appointments, self-monitoring by means of paper diaries, and weekly group meetings were also examples of the content of control groups. There are various ways to implement weight loss interventions that use technology. It is not known which features are imperative for achieving weight loss [35], but this review provides a general view of the features that were used in previous studies as well as the minimum and maximum features that were used in previous studies.

A challenge of technology-based interventions might be how to motivate participants to use technology enough. For example, in the study by Hansen et al [70], 71% of the participants did not sign into the website at all, and only 2% signed in several times over a 6-month period. Haapala et al [85] found that the frequency of using the website varied from 3 to 8 times per week. According to the investigators, the participants who achieved a weight loss of >5% reported more log-ins to the website than the participants who achieved a weight loss of <5%. To achieve results using weight loss interventions, it would be important to get participants to engage in the study. In future studies, this could be a covariate because it is an important element of weight loss interventions. Future studies should more accurately report the adherence of participants to the intervention.

Outcome variables have varied in previous reviews concerning body composition changes. Therefore, the clinical significance of the reduction in BMI, waist circumference, or body fat percentage is difficult to determine. There are also certain challenges in these outcomes. BMI has been used to determine obesity. However, it is a poor indicator of body fat percentage, and it does not capture the location of body fat [100]. A clinically significant reduction in waist circumference has not been determined in previous studies. Han et al [101] found that a 5-10 cm reduction in waist circumference was a realistic goal with great health benefits for White women who were overweight. However, this study was published in 1997, and it is out of date. Future studies are needed to determine what a beneficial reduction in waist circumference is in terms of, for example, the prevention of obesity-related illnesses. Body fat percentage can be measured in multiple ways. In this review, 23% (7/30) of the studies used bioimpedance [64,72,73,76,78,89,93], 3% (1/30) used DXA [86], and 3% (1/30) used skinfold [80]. In 7% (2/30) of the studies, the methods were not mentioned [70,92]*.* Benito et al [8] compared DXA, bioimpedance, and skinfold measurements in adults who were overweight. According to them, skinfold seems to underestimate the values of body fat percentage. Therefore, DXA and bioimpedance can be better tools for measuring it [8]. Liao et al [102] studied the accuracy and agreement of DXA and bioimpedance results. They found moderate to high correlations between these 2 measurements in estimating total and segmental lean body mass, fat mass, and body fat percentage.

Obesity is a multidimensional phenomenon, and although obesity is a widely studied condition, we still do not understand all aspects of it. Strong misconceptions such as laziness and lack of self-discipline are still related to obesity in health care settings, workplaces and education, and public literature. In addition, people with obesity feel a strong stigma, which can affect their physical and psychological well-being [20]. It is important to understand comprehensively the phenomenon of obesity so that in rehabilitation settings we can support the well-being of people with obesity without stigmatization.

### Strengths and Limitations

The strength of this systematic review is its carefully conducted statistical analyses with its strict PICOS criteria and the use of only RCTs. Meta-regression analysis with several covariates was used to explain the heterogeneity of the studies. To the best of our knowledge, this is the first systematic review to use a comprehensive analysis to identify possible statistical heterogeneity while investigating the effectiveness of web- and mobile-based distance weight loss interventions in rehabilitation settings on body composition. A limitation of this systematic review was that the included studies were very heterogeneous. An explanation for the clinical heterogeneity might be the variability in the content of the interventions.

Despite several covariates used in the meta-regression analysis, only personal feedback showed a statistically significant association, and the quality of the studies showed a nonsignificant association with the reduction in waist circumference and body fat percentage. In addition, there was variation in the widths of the CIs, and all the estimates were small. It is also important to note that no multiple testing corrections were made. In addition, there was low statistical power. Therefore, any conclusions drawn from the results of the meta-regression analysis must be treated with caution. According to our findings, the sources of the heterogeneity remain unclear. Some other factors that were not investigated in this study could probably explain the heterogeneity. For example, previous systematic reviews observed associations between social support and waist circumference reduction [33] and between program use and weight loss [61].

The second limitation is that only 1 researcher screened the studies. However, the whole study group supported the study screening, and in unclear cases, the study group was consulted. In the final search, 30 studies met the inclusion criteria; therefore, the review provides a comprehensive view of the topic in question. Third, this review focuses on web- and mobile-based weight loss rehabilitation interventions, which were mostly implemented remotely by a health care professional. Because of this, we cannot generalize the findings of this review to all persons and weight loss interventions. It is possible that other factors in the rehabilitation context, such as social support provided by a health care professional, may have affected the findings. Despite these limitations, we believe that the important findings of this review provide a unique overview of web- and mobile-based distance weight loss interventions to clinicians and researchers in rehabilitation settings.

In this review, 87% (26/30) of the studies were from high-income western countries. Only 13% (4/30) of the studies were from Asia [67,73,79,83]; the rest were from the United States, Australia, or Europe. Because of the high representation of high-income western countries, we were not able to perform a meta-regression analysis for identifying differences across continents. Therefore, the findings of our results cannot be generalized worldwide and more future studies are recommended to investigate the potential of, and possible differences in, the use of technology to support weight loss and to reduce obesity in different cultures.

### Future Recommendations

Future studies should focus on the comparability of the intervention content. To study weight loss more specifically, more attention should be paid to the comparability of RCTs and control group content. Studies should focus on interventions where the only difference between the experimental and control groups would be the use of technology; in other respects, the content of the groups would be similar. With this study design, it would be possible to determine the effect of technology in a weight loss intervention. In our review, the content of the interventions in the included studies was heterogeneous, and this may influence the generalizability of our findings. However, this review provides a glance at technology-based interventions in weight loss rehabilitation. Future studies should use the CONSORT-EHEALTH (Consolidated Standards of Reporting Trials of Electronic and Mobile Health Applications and Online Telehealth) checklist [103] to align the terminology for the use of technology and, in turn, increase the quality of future systematic reviews.

In our meta-regression analysis, we considered different aspects of the intervention content to explain the statistical heterogeneity. A study concerning the remote rehabilitation of cardiac rehabilitees’ by means of technology [104] suggested that interventions are encouraged to include social participation, such as peer group discussion and personalized feedback. In our review, we were unable to include such covariates to describe the role of communication because only 7% (2/30) of the studies [56,78] used 2-way communication and only 17% (5/30) of the studies included peer support [68,70,74,79,81] through technology. Other studies included a 1-way communication approach. Although we could not use communication as a covariate, we used personal feedback as a covariate. It had an impact on BMI but not on waist circumference or body fat percentage. Future studies should consider communication as an essential tool in supporting the weight loss of participants.

Future studies are also needed to better understand weight loss and identify which components are essential in achieving it. Several covariates did not explain the variability of the results, which may indicate that web- and mobile-based distance interventions in rehabilitation settings in the home environment may be more complex to study and require a more personalized approach. Therefore, in the future, it would be recommended to study the meanings and perceptions of participants on the use of technology in distance rehabilitation settings. As in the cardiac rehabilitees’ technology study [104], more personalized approaches should be used in weight loss interventions. Rehabilitation is a complex trust-building process in which, for example, cardiac rehabilitees desire personalized and individualized counseling to maintain motivation [105]. Although weight loss has been widely studied, we still do not understand its phenomena. An aspect that is closely related to personalization is the understanding of obesity-related stigma, which affects the well-being of people with obesity [20]. Future studies should investigate the experiences of stigma and increase the awareness of obesity-related factors as well as our understanding of the stigma for people with obesity.

### Conclusions

Web- and mobile-based distance weight loss interventions might be more effective than weight loss interventions without the use of technology. However, the changes in body composition outcomes in this review were not clinically meaningful, and statistical and clinical heterogeneity were present. Future studies are needed to better understand weight loss and identify what components are essential in achieving it.

## Appendix

Multimedia Appendix 1 An example of the search strategy.

Multimedia Appendix 2 Description of the included studies.

Multimedia Appendix 3 Quality points of the included studies.

Multimedia Appendix 4 Results of the meta-regression analysis of BMI, waist circumference, and body fat percentage.

Multimedia Appendix 5 Main results of the studies included in the meta-analysis of BMI.

Multimedia Appendix 6 Main results of the studies included in the meta-analysis of waist circumference.

Multimedia Appendix 7 Main results of the studies included in the meta-analysis of body fat percentage.

## Abbreviations

AICc: corrected Akaike information criterion
CONSORT-EHEALTH: Consolidated Standards of Reporting Trials of Electronic and Mobile Health Applications and Online Telehealth
DXA: dual-energy x-ray absorptiometry
MD: mean difference
PICOS: Population, Intervention, Comparison, Outcomes, and Study Design
PRISMA: Preferred Reporting Items for Systematic Reviews and Meta-Analyses
RCT: randomized controlled trial
