# Supplementary material for: The Effectiveness of Physical Activity-Promoting Web- and Mobile-Based Distance Weight Loss Interventions on Body Composition in Rehabilitation Settings: Systematic Review, Meta-analysis, and Meta-Regression Analysis
Source: J Med Internet Res. 2022 Mar 24;24(3):e25906. doi: 10.2196/25906 (PMC8990343; doi:10.2196/25906)
Supplement: Multimedia Appendix 1 [file jmir_v24i3e25906_app1.doc]

Multimedia Appendix 1.

An example of a search strategy

Database: Ovid MEDLINE(R) <1946 to October Week 1 2014>

Search Strategy:

--------------------------------------------------------------------------------

1 Exercise Therapy/ (27416)

2 exercise therapy.tw. (1900)

3 Physical Therapy Modalities/ (28897)

4 physical therapy.tw. (10161)

5 physiotherapy.tw. (10909)

6 functional therapy.tw. (295)

7 Occupational Therapy/ (10498)

8 Neuropsychology/ (2044)

9 dietician.tw. (576)

10 dietitian.tw. (1805)

11 Dietetics/ (5248)

12 Occupational Health Services/ (9835)

13 multidisciplinary therapy.tw. (270)

14 physical activity.tw. (55661)

15 Exercise/ (70603)

16 Exercise Movement Techniques/ (418)

17 Motor Activity/ (83150)

18 energy expenditure.tw. (17234)

19 "Delivery of Health Care"/ (68140)

20 public health service$.tw. (5236)

21 Nursing Diagnosis/ (3863)

22 Nursing Informatics/ (1017)

23 Community Health Nursing/ (18608)

24 Nursing/ (50228)

25 Public Health Nursing/ (9754)

26 medical treatment$.tw. (34601)

27 Psychiatry/ (32921)

28 Rehabilitation/ (17036)

29 Health Promotion/ (55591)

30 health counse?ling.tw. (556)

31 directive counse?ling.tw. (128)

32 coaching.tw. (2284)

33 health guidance.tw. (273)

34 "Activities of Daily Living"/ (52849)

35 adl.tw. (6077)

36 participation.tw. (91377)

37 cultural activities.tw. (158)

38 Leisure Activities/ (6678)

39 "Physical Education and Training"/ (12075)

40 Primary Prevention/ (14663)

41 Secondary Prevention/ (2154)

42 Tertiary Prevention/ (87)

43 Sports/ (24021)

44 active lifestyle.tw. (816)

45 physical lifestyle.tw. (27)

46 Physical Fitness/ (22813)

47 Health Education/ (53678)

48 Patient Education as Topic/ (72468)

49 Behavior Therapy/ (24576)

50 Cognitive Therapy/ (17151)

51 or/1-50 (863030)

52 mobile system$.tw. (153)

53 Telemedicine/ (12179)

54 ehealth.tw. (644)

55 mobile health.tw. (424)

56 mhealth.tw. (184)

57 phealth.tw. (35)

58 mobile multimedia.tw. (10)

59 mobile communication$.tw. (402)

60 mobile technolog$.tw. (353)

61 Cellular Phone/ (4868)

62 cellular phone$.tw. (551)

63 cell phone$.tw. (1154)

64 cellular telephone$.tw. (335)

65 mobile phone$.tw. (2761)

66 mobile telephone$.tw. (334)

67 Mobile Health Units/ (3053)

68 Computers, Handheld/ (2492)

69 communication technolog$.tw. (1596)

70 technology integration.tw. (67)

71 web based communication$.tw. (58)

72 web based organi?ation$.tw. (0)

73 virtual communit$.tw. (158)

74 e-learning environment$.tw. (26)

75 User-Computer Interface/ (30035)

76 virtual learning environment$.tw. (118)

77 acceleromet$.tw. (6771)

78 mobile application$.tw. (164)

79 web based interacti$.tw. (126)

80 (mobile adj3 game$).tw. (22)

81 mobile gaming.tw. (3)

82 pervasive game$.tw. (0)

83 Geographic Information Systems/ (4803)

84 global positioning system$.tw. (721)

85 telerehabilitation.tw. (218)

86 tele rehabilitation.tw. (40)

87 "web 2.0 intervention$".tw. (4)

88 "web 2.0 application$".tw. (29)

89 smart phone$.tw. (207)

90 Remote Consultation/ (3752)

91 sms.tw. (2922)

92 Text Messaging/ (666)

93 text messag$.tw. (918)

94 digital learning.tw. (21)

95 or/52-94 (71324)

96 Randomized Controlled Trials as Topic/ (98775)

97 Randomized Controlled Trial/ (396594)

98 Random Allocation/ (83641)

99 Double-Blind Method/ (131663)

100 Single-Blind Method/ (20356)

101 Clinical Trial/ (499445)

102 clinical trial, phase i.pt. (15178)

103 clinical trial, phase ii.pt. (24340)

104 clinical trial, phase iii.pt. (9914)

105 clinical trial, phase iv.pt. (1010)

106 controlled clinical trial.pt. (90437)

107 randomized controlled trial.pt. (396594)

108 multicenter study.pt. (186489)

109 clinical trial.pt. (499445)

110 exp Clinical Trials as Topic/ (293077)

111 or/96-110 (1081783)

112 (clinical adj trial$).tw. (213285)

113 ((signl$ or doubl$ or treb$ or tripl$) adj (blind$3 or mask$3)).tw. (118789)

114 Placebos/ (33931)

115 placebo$.tw. (158807)

116 randomly allocated.tw. (16408)

117 (allocated adj2 random$).tw. (18923)

118 or/112-117 (411822)

119 111 or 118 (1209420)

120 case report.tw. (189105)

121 letter/ (845830)

122 Historical Article/ (311099)

123 or/120-122 (1334329)

124 119 not 123 (1178568)

125 51 and 95 and 124 (2193)

126 limit 125 to (yr="2000 -Current" and ("adult (19 to 44 years)" or "middle age (45 to 64 years)") and (english or finnish or german or swedish) and humans) (1238)

127 intervention studies/ (7408)

128 intervention$.tw. (541118)

129 127 or 128 (542366)

130 126 and 129 (681)
