# Supplementary material for: The Effectiveness of Physical Activity-Promoting Web- and Mobile-Based Distance Weight Loss Interventions on Body Composition in Rehabilitation Settings: Systematic Review, Meta-analysis, and Meta-Regression Analysis
Source: J Med Internet Res. 2022 Mar 24;24(3):e25906. doi: 10.2196/25906 (PMC8990343; doi:10.2196/25906)
Supplement: Multimedia Appendix 2 [file jmir_v24i3e25906_app2.doc]

Multimedia Appendix 2. Description of the included studies

| **Research / Year/ Country** | **Duration and adherence**  **(%)** | **N**  **(%men)** | **Experimental**  **N**  **(%men)** | **Control**  **N**  **(% men)** | **Age intervention/ control (year) Mean (SD)** | **Participants**  **description** | **Intervention and intensity** | **Control** | **Variables** | |
| --- | --- | --- | --- | --- | --- | --- | --- | --- | --- | --- |
| **Primary prevention** | | | | | | | | | | |
| **Chambliss et al. [45]**  **2010**  **USA** | 12 weeks | 120 (17.1) | Basic: 45 (17.8)  Enhanced: 45 (20.0) | 30 (13.3) | Basic: 45.1 (10.3) Enhanced: 46.1 (9.4) Control: 43.2 (11.5) | Overweight adults | Basic: meeting with the health educator, individual report of measurements, 1 h group seminar, basic guidance for healthy eating and physical activity, self-monitoring daily food intake and physical activity with the software program, weekly tailored reports about the diaries, monthly measurement visits and discussion of treatment goals and progress  Enhanced: all components from the basic group. In addition, they received 2 h seminar, step counters, monthly email newsletters, brief monthly telephone consultations, weekly behavioral tracking forms which were returned via email and supplemented the computer-generated reports allowing the health educator to provide tailored feedback, monthly clinic visits | Wait-list control group | Body mass index and waist circumference | |
| **Collins et al. [47] 2012**  **Australia** | 12 weeks | 309 (42) | Basic: 99 (41)  Enhanced: 106 (42) | 104 (42) | 42 (10.2) | Overweight and obese adults | Basic: free access to the basic Web-based program  Enhanced: enhanced version of the Web-based program plus personalized reports, e-feedback and an escalating reminder schedule | A wait-list control group | Waist circumference and body mass index | |
| **Haapala et al. [64]**  **2009**  **Finland** | 12 months | 82 (22.5) | 42 (21) | 40 (24) | 38.1 (4.7) | Healthy overweight adults | Mobile phone-operated weight-loss program and a webspace for dietary record keeping, weight loss tacking in visual form and links | No intervention, offered the studied weight-loss program after the 12-month visit | Waist circumference | |
| **Hansen et al. [49]**  **2012**  **Denmark** | 3 months +  3 months follow-up | 1168  (42) | 583 (40.8) | 585 (43.2) | 50 (13.6) | Physically inactive adults | Access to a physical activity website, where was 1) a personal page, 2) a page with training programs and general recommendations and 3) a forum and discussion page where physiotherapist answered to questions | No intervention | Body mass index and body fat percentage | |
| **Huber et al. [50] 2015**  **USA** | 3 months + 3 months follow-up | 77 (20.7) | 38 (16) | 39 (36) | 47.9 (13.2) | Obese patients in a primary care setting | Portion control plate and telecoaching | Institutional pamphlets on healthy eating and exercise habits | Waist circumference and body mass index | |
| **Hunter et al. [51] 2008**  **USA** | 6 months | 446 (49.8) | 224  (50.0) | 222  (49.5) | 34.0 (7.3) | A diverse sample of primarily overweight adults (The U. S. Air Force) | Behavioral Internet treatment (food and exercise diaries, personalized feedback and lessons on the website), two motivational interviewing telephone calls, in-person orientation and the same as the usual care group | Meeting with the primary care provider annually, one fitness center, weight loss and healthy cooking classes, nutrition and fitness assessments and testing for fitness, BMI and waist-circumference standards | Change in percent body fat, body mass index and waist circumference | |
| **Lin et al. [52]**  **2014**  **China** | 6  months | 110  (39.8) | 56 (39.7) | 54 (40) | 38.4 (8.0) / 38.1 (8.1) | Overweight adults in Beijing | Personalized behavior change goals, self-monitoring their adherence to these goals via a daily text message, three group sessions, pedometer and five coaching calls | Brief information session | Waist circumference, body fat percent and body mass index | |
| **Mehring et al. [53]**  **2013**  **Germany** | 12 weeks | 133 (33.9) | 73  (30.3) | 60 (37.5) | 46.5 (10.9) / 50.9 (15.3) | Overweight individuals of whom a weight reduction was recommendable | Individualized education, motivation, exercise guidance, daily SMS reminder, self-monitoring via Internet and 3 telephone calls | Advised by the general practitioner in their individual way of usual care to reduce weight | Difference in waist circumference and body mass index | |
| **Melchart et al. [54]**  **2017**  **Germany** | 12 months | 166 (25.9) | 111 (33.7) | 55 (27.3) | 49.9 (9.7) / 52.1 (10.0) | Overweight persons (BMI 25–30)  or those with grade 1 obesity (BMI 30–35) | The first 3 months: 3 full-day “introduction days” plus 10  2-hour weekly training sessions; and 2) during the following 9 months, participants  practice lifestyle modifications by themselves, supported  by lifestyle counseling via telephone or email. The program encompasses access to a web-based health  portal, providing detailed advice and instructions with respect to food, exercise, and relaxation. | Common practice in UC with standard advice from a doctor. A leaflet containing 10 acknowledged rules for healthy food and physical exercise according to the German Nutrition Society. | Body mass index and waist circumference | |
| **Morgan et al. [55]**  **2012**  **Australia** | 3 months + 3 months follow-up | 159 (100) | SHED-IT Resource: 54 (100)  SHED-IT Online: 53 (100) | 52 (100) | 47.5 (11.0) | Overweight and obese men | SHED-IT Resources: weight loss resource package (Weight Loss DVD for Blokes, Weight Loss Handbook for Blokes, Weight Loss Support Book for Blokes, pedometer, tape measure for waist circumference and kilojoule counter book)  SHED-IT Online group: Same materials plus website user guide and online diary and feedback emails | Wait-list control, no intervention | Waist circumference and Body composition (body fat percent, visceral fat area, skeletal muscle mass) and body mass index | |
| **Rogers et al. [57] 2015**  **USA** | 6 months | 39 (20.5) | EN-TECH: 13 (23.1)  TECH: 12 (25) | SBWL: 14 (14.3) | EN-TECH: 39.5 (9.7) / TECH: 40.6 (9.5) / SBWL: 39.7 (11.5) | Adults with obesity | Technology-based system: The BodyMedia® FIT System in self-monitoring, a wearable device that monitored physical activity and energy expenditure, one in-person session and a telephone calls once per month  Enhanced technology-based system: the same intervention  as TECH plus an enhanced technology-based system (Bluetooth® capability allowed calories and physical activity transmitting to a smartphone app | Weekly group  meetings, self-monitoring of dietary intake and physical activity with the use of a paper diary | Waist circumference, body mass index and body fat percent | |
| **Sakane et al. [58] 2013**  **Japan** | 12 weeks | 270 (100) | Web: 95 (100)  Web + VFA: 88 (100) | 87 (100) | − | Overweight and obese men | Web: Web-based weight-loss program  Web + VFA: Web plus visceral fat measurement and education focused on increased health risks associated with visceral fat accumulation | − | Change in waist circumference and body mass index | |
| **Shuger et al. [59] 2011**  **USA** | 9 months | 197 (18.3) | GWL: 49 (16)  SWA: 49 (18.4)  GWL+SWA: 49 (18.4) | 50 (16) | GWL: 46.8 (12.4) SWA: 47.7 (11.6)  GWL+SWA: 45.7 (10.4)  Standard care: 47.2 (8.9) | Sedentary overweight or obese adults | The evidence-based weight loss manual  GWL: a 14-week group-based behavioral weight loss program followed by weekly, biweekly, and monthly telephone counseling calls  SWA-alone: the armband and access to a web account  GWL+SWA: the group-based behavioral weight loss program and follow-up telephone counseling calls plus the armband | A self-directed weight loss program with an evidence-based weight loss manual | Waist circumference, body mass index and percent body fat | |
| **Stephens et al. [60]**  **2017**  **USA** | 3 months | 62 (29.0) | 31 (25.9) | 31 (32.3) | 20.0 (range 18-24) / 20.0 (range 18-25) | Individuals between 18 and 25 years of age with a BMI between 25 and 40 kg/m2 who owned an iPhone or Android phone | 30- to- 40-minute counseling session, the Lose it! application and individualized text messages | Asked not to use any smartphone  applications focused on weight loss for the duration of  the study. At 3-month visit they received the Lose It! application with a  training session | Waist circumference and body mass index | |
| **van Wier et al. [70]**  **2009**  **Netherlands** | 6 months | 702 (67) | Phone: 236 (69.5)  Internet: 235 (65.1) | 231 (66.5) | 43 (8.6) | Overweight working population | Self-help materials and lifestyle intervention program which included 10 modules.  Phone group: After each module, personal counsellors contacted them by phone  Internet group: After each module, personal counsellors contacted them by e-mail. Access to an interactive website | Self-help materials, no counseling | Change in waist circumference | |
| **Secondary and tertiary prevention** | | | | | | | | | | |
| **Aguiar et al. [43]**  **2016**  **Australia** | 6 months | 101  (100) | 53  (100) | 48  (100) | 52.5 (9.5) / 52.2 (10.1) | Men with high risk for  developing type 2 diabetes | An individual orientation, The PULSE Program resource pack (Weight Loss Program -pedometer, self-reporting, DVD, book and booklet-, Diabetes Prevention Handbook and The Exercise Support Book) | The waitlist control group received the PULSE program after their 6-month control period | Waist circumference, body mass index and body fat percent | |
| **Anderson et al. [44]**  **2010**  **USA** | 12 months | 295 (42.0) | 146 (41.1) | 149 (43) | ‒ | Patients with type 2 diabetes | Telephonic disease management | Continued to receive primary care at  Community Health Center | Body mass index | |
| **Chen et al. [62]**  **2012**  **Taiwan** | 3 months | 63  (0) | 31 (0) | 32 (0) | 41.9 (9.8) /  45.7 (8.3) | Career women with metabolic syndrome risk factors | Internet-based health management platform (a health examination database, nutrition management system and exercise management system) | No intervention | Waist circumference | |
| **Cho et al. [46]**  **2017**  **Korea** | 4 weeks | 47 (89.4) | 24 (83) | 23 (96) | 42.0 (8.0) / 44.0 (11.0) | Adults with witnessed snoring or sleep apnea | Participants were educated to modify their lifestyle to lose weight by using a smartphone-based app. The app is composed of two main modules: a diet and a physical activity module | Verbally educated to modify their lifestyle to lose weight | Body mass index | |
| **Devi et al. [71]**  **2014**  **UK** | 6 weeks + 6 month follow-up | 80  (74) | 39 (71) | 41 (78) | 66.3 (8.4) / 66.2 (10.1) | Patients with chronic stable angina | Web-based rehabilitation program, online exercise diary and communication with rehabilitation specialists with email link/synchronized chat room | Treatment with general practitioner as usual | Body fat percentage | |
| **Eakin et al. [63] 2014**  **Australia** | 18  months + follow-up in 24 months | 252  (56.3) | 121  (55.6) | 131  (57) | 57.7 (8.1) / 58.3 (9.0) | Primary care patients with type 2 diabetes | Telephone counseling and pedometer | Usual care | Waist circumference | |
| **Hageman et al. [48]**  **2014**  **USA** | 24 months | 270  (0) | Web-based: 106 (0)  Print-mailed: 111 (0) | 53 (0) | 56.4 (6.3) | Women with prehypertension | Two 2-hour training sessions, home blood pressure monitor, pedometer and 5 phone call sessions, resistance bands and instructional exercise videos  Web-based: Access to the web-page for self-monitoring eating, activity and blood pressure, 18 web-based newsletters  Print-mailed: Self-monitoring eating, activity and blood pressure using paper logs, 18 print-mailed newsletters | One 30-minute introductory education session and printed educational materials | Waist circumference and body mass index | |
| **Harrigan et al. [65]**  **2016**  **USA** | 6 months | 100 (0) | In-person: 33 (0)  Telephone: 34 (0) | 33 (0) | 59.0 (7.5) | Breast cancer survival | In-person: individualized counseling sessions, LEAN book (self-reporting), home-based physical activity program and pedometer  Telephone: Same intervention except counseling sessions were telephone-based | Brochures and two session weight management program. At the end of the study, they were offered the LEAN book and LEAN Journal. | Waist circumference and body fat | |
| **Karhula et al. [66]**  **2015**  **Finland** | 12 months | 425 (61.1) | Heart disease:  160 (65.3)  Diabetes:  143 (55) | Heart disease:  65 (68)  Diabetes:  57 (57) | 69.1 (9.1) | Patients with type 2 diabetes or heart disease | Phone calls from the health coach, health coaching over mobile phones and self-monitoring of health parameters with the help of a remote patient monitoring system, a toolbox (a mobile phone, a mobile personal health record app and a set of measurement devices) | Disease management information booklet, laboratory tests taken once a year and one appointment or phone call by a nurse or doctor. | Waist circumference | |
| **Ligibel et al. [67] 2012**  **USA** | 16  weeks | 99  (7.4) | 48  (8.0) | 51  (7.0) | 53.1 (10.8) / 55.5 (10.6) | Cancer survivor adults | 10 phone calls and a pedometer | Routine care and the possibility to receive a telephone consultation from an exercise trainer after the intervention | Waist circumference | |
| **Matthews**  **et al. [72]**  **2006**  **USA** | 12 weeks | 36 (0) | 22 (0) | 14 (0) | 51.3 (9.0) / 56.9 (12.3) | Women with early stage breast cancer | A single in-person counseling visit, five telephone-counseling calls and a pedometer | Usual care, “keep the normal level of PA” | Percent body fat | |
| **Reeves et al. [68] 2017**  **Australia** | 6 months | 90  (0) | 45  (0) | 45  (0) | 56.4 (9.0) / 54.3 (8.4) | Women who recently completed  treatment for breast cancer with a BMI 25–40 kg/m2 | 16 telephone calls, workbook, self-monitoring diary, digital scales, pedometer and caloriecounter | Mailed feedback following  their baseline and 6-month assessment. After 6-month the intervention workbook and diary | Waist circumferences and body fat percent | |
| **Rimmer et al. [56]**  **2013**  **United States** | 9  months | 91  (24.2) | POWERS: 32  (28.1)  POWERS(plus): 32  (22.2) | 38  (21.9) | POWERS: 46.2 (14.0)  POWERS(plus):44.3 (12.8)  C: 48.8 (11.1) | Adults with physical  disabilities (spinal cord injury, multiple sclerosis, spina bifida, cerebral palsy, stroke, or lupus) | Telephone-based weight management program  using a Web-based system  POWERS: physical activity only  POWERS(plus): physical activity plus nutrition  a physical activity tool kit and regular coaching telephone calls | The physical activity tool kit and self-guided health promotion  resources at the completion of the trial but no coaching | Body mass index | |
|  |  |  |  |  |  |  |  |  |  | |
| **Stuart et al. [69] 2012**  **Australia** | 12 weeks | 49 (38.8) | 26 (34.6) | 23 (43.5) | 48.0 (5.9) | Patients at risk of developing cardiovascular disease | Comprehensive lifestyle intervention program (CLIP): 7 telephone calls, a CLIP handbook (dietary guide, sample menu plans and recipes). At week 4 participants received a copy of the CSIRO Healthy Heart Programme which contain aerobic exercise component and at week 6, a muscle-strengthening program | Usual care from the general practitioner and general printed lifestyle materials | Waist circumference |  |
| **Watson et al. [61]**  **2015**  **UK** | 12 months | 65 (44.6) | 32 (50.0) | 33 (61.0) | 51.4 (7.6) / 52.9 (7.3) | Adults with 1 or more cardiovascular disease risk factor | Web-based program, Imperative Health package that contained the self-monitoring devices (Bluetooth-enabled  weighing scales and an accelerometer activity band) | Usual self  and medical care | Waist circumference and body mass index |  |
