# Supplementary material for: The Effectiveness of Physical Activity-Promoting Web- and Mobile-Based Distance Weight Loss Interventions on Body Composition in Rehabilitation Settings: Systematic Review, Meta-analysis, and Meta-Regression Analysis
Source: J Med Internet Res. 2022 Mar 24;24(3):e25906. doi: 10.2196/25906 (PMC8990343; doi:10.2196/25906)
Supplement: Multimedia Appendix 3 [file jmir_v24i3e25906_app3.doc]

Multimedia Appendix 3. The quality points of the included studies (N=30).

| Study and year | 1: Randomization method adequate | 2: Treatment allocation concealed | 3: Blinding of participants | 4: Blinding of care provider | 5: Blinding of outcome assessor | 6: Dropouts described and acceptable | 7: Participants analyzed in the allocated groups | 8: Free of suggestion of selective outcome reporting | 9: Group similarity at the baseline | 10: Cointervention avoided or similar | 11: Compliance | 12: Similar timing of the outcome assessment | 13: Other sources of potential bias unlikely | Number of *Yes* scores (maximum of 13) |
| --- | --- | --- | --- | --- | --- | --- | --- | --- | --- | --- | --- | --- | --- | --- |
| Aguiar et al. [64], 2016 | Yes^a^ | Yes^a^ | No^b^ | ? ^c^ | Yes^a^ | Yes^a^ | Yes^a^ | Yes^a^ | Yes^a^ | ? ^c^ | No^b^ | Yes^a^ | Yes^a^ | 9 |
| Anderson et al. [65], 2010 | Yes^a^ | ? ^c^ | ? ^c^ | ? ^c^ | ? ^c^ | No^b^ | Yes^a^ | Yes^a^ | Yes^a^ | ? ^c^ | Yes^a^ | Yes^a^ | Yes^a^ | 7 |
| Chambliss et al. [66], 2010 | Yes^a^ | ? ^c^ | ? ^c^ | ? ^c^ | ? ^c^ | No^b^ | Yes^a^ | Yes^a^ | Yes^a^ | Yes^a^ | Yes^a^ | Yes^a^ | Yes^a^ | 8 |
| Chen et al. [83], 2014 | ? ^c^ | ? ^c^ | No^b^ | ? ^c^ | ? ^c^ | Yes^a^ | ? ^c^ | ? ^c^ | Yes^a^ | ? ^c^ | ? ^c^ | Yes^a^ | Yes^a^ | 4 |
| Cho et al. [67], 2018 | ? ^c^ | ? ^c^ | ? ^c^ | ? ^c^ | ? ^c^ | No^b^ | Yes^a^ | Yes^a^ | Yes^a^ | ? ^c^ | Yes^a^ | Yes^a^ | Yes^a^ | 6 |
| Collins et al. [68], 2012 | Yes^a^ | Yes^a^ | Yes^a^ | ? ^c^ | Yes^a^ | No^b^ | Yes^a^ | Yes^a^ | Yes^a^ | Yes^a^ | ? ^c^ | Yes^a^ | Yes^a^ | 10 |
| Devi et al. [92], 2014 | Yes^a^ | Yes^a^ | No^b^ | ? ^c^ | No^b^ | Yes^a^ | ? ^c^ | ? ^c^ | Yes^a^ | Yes^a^ | No^b^ | Yes^a^ | Yes^a^ | 7 |
| Eakin et al. [84], 2014 | Yes^a^ | Yes^a^ | ? ^c^ | ? ^c^ | Yes^a^ | Yes^a^ | Yes^a^ | Yes^a^ | Yes^a^ | ? ^c^ | Yes^a^ | Yes^a^ | Yes^a^ | 10 |
| Haapala et al. [85], 2009 | ? ^c^ | ? ^c^ | No^b^ | ? ^c^ | Yes^a^ | Yes^a^ | No^b^ | ? ^c^ | Yes^a^ | No^b^ | ? ^c^ | No^b^ | Yes^a^ | 4 |
| Hageman et al. [69], 2014 | Yes^a^ | Yes^a^ | No^b^ | ? ^c^ | Yes^a^ | Yes^a^ | No^b^ | No^b^ | No^b^ | Yes^a^ | ? ^c^ | Yes^a^ | Yes^a^ | 7 |
| Hansen et al. [70], 2012 | Yes^a^ | ? ^c^ | No^b^ | ? ^c^ | Yes^a^ | No^b^ | No^b^ | Yes^a^ | Yes^a^ | ? ^c^ | No^b^ | Yes^a^ | Yes^a^ | 6 |
| Harrigan et al. [86], 2016 | Yes^a^ | Yes^a^ | No^b^ | ? ^c^ | ? ^c^ | ? ^c^ | No^b^ | ? ^c^ | Yes^a^ | ? ^c^ | Yes^a^ | Yes^a^ | Yes^a^ | 6 |
| Huber et al. [71], 2015 | Yes^a^ | Yes^a^ | No^b^ | No^b^ | No^b^ | No^b^ | No^b^ | ? ^c^ | No^b^ | ? ^c^ | Yes^a^ | Yes^a^ | Yes^a^ | 5 |
| Hunter et al [72], 2008 | Yes^a^ | No^b^ | No^b^ | No^b^ | No^b^ | Yes^a^ | Yes^a^ | No^b^ | Yes^a^ | ? ^c^ | ? ^c^ | Yes^a^ | Yes^a^ | 6 |
| Karhula et al. [87], 2015 | Yes^a^ | ? ^c^ | No^b^ | ? ^c^ | ? ^c^ | Yes^a^ | No^b^ | No^b^ | Yes^a^ | ? ^c^ | Yes^a^ | Yes^a^ | Yes^a^ | 6 |
| Ligibel et al. [88], 2012 | ? ^c^ | ? ^c^ | ? ^c^ | ? ^c^ | ? ^c^ | Yes^a^ | Yes^a^ | Yes^a^ | Yes^a^ | ? ^c^ | Yes^a^ | Yes^a^ | Yes^a^ | 7 |
| Lin et al. [73], 2014 | Yes^a^ | ? ^c^ | ? ^c^ | ? ^c^ | Yes^a^ | Yes^a^ | No^b^ | ? ^c^ | Yes^a^ | ? ^c^ | Yes^a^ | Yes^a^ | Yes^a^ | 7 |
| Matthews et al. [93], 2006 | Yes^a^ | ? ^c^ | ? ^c^ | ? ^c^ | ? ^c^ | ? ^c^ | Yes^a^ | ? ^c^ | Yes^a^ | ? ^c^ | Yes^a^ | Yes^a^ | Yes^a^ | 6 |
| Mehring et al. [74], 2013 | Yes^a^ | ? ^c^ | ? ^c^ | ? ^c^ | ? ^c^ | ? ^c^ | No^b^ | ? ^c^ | No^b^ | ? ^c^ | ? ^c^ | Yes^a^ | Yes^a^ | 3 |
| Melchart et al. [75], 2017 | Yes^a^ | ? ^c^ | ? ^c^ | ? ^c^ | ? ^c^ | Yes^a^ | No^b^ | Yes^a^ | Yes^a^ | ? ^c^ | Yes^a^ | Yes^a^ | Yes^a^ | 7 |
| Morgan et al. [76], 2012 | Yes^a^ | Yes^a^ | No^b^ | ? ^c^ | Yes^a^ | Yes^a^ | Yes^a^ | Yes^a^ | Yes^a^ | Yes^a^ | No^b^ | Yes^a^ | Yes^a^ | 10 |
| Reeves et al. [89], 2017 | Yes^a^ | Yes^a^ | No^b^ | ? ^c^ | Yes^a^ | Yes^a^ | Yes^a^ | Yes^a^ | No^b^ | ? ^c^ | Yes^a^ | Yes^a^ | Yes^a^ | 9 |
| Rimmer et al. [77], 2013 | Yes^a^ | ? ^c^ | ? ^c^ | ? ^c^ | Yes^a^ | Yes^a^ | Yes^a^ | ? ^c^ | Yes^a^ | ? ^c^ | ? ^c^ | Yes^a^ | Yes^a^ | 7 |
| Rogers et al. [78], 2015 | ? ^c^ | ? ^c^ | ? ^c^ | ? ^c^ | ? ^c^ | No^b^ | No^b^ | ? ^c^ | Yes^a^ | Yes^a^ | ? ^c^ | Yes^a^ | ? ^c^ | 3 |
| Sakane et al. [79], 2013 | ? ^c^ | ? ^c^ | ? ^c^ | ? ^c^ | Yes^a^ | ? ^c^ | Yes^a^ | ? ^c^ | ? ^c^ | ? ^c^ | Yes^a^ | Yes^a^ | Yes^a^ | 5 |
| Shuger et al. [80], 2011 | Yes^a^ | ? ^c^ | No^b^ | No^b^ | Yes^a^ | No^b^ | Yes^a^ | Yes^a^ | Yes^a^ | ? ^c^ | ? ^c^ | Yes^a^ | Yes^a^ | 7 |
| Stephens et al. [81], 2017 | Yes^a^ | ? ^c^ | No^b^ | ? ^c^ | ? ^c^ | Yes^a^ | ? ^c^ | ? ^c^ | Yes^a^ | Yes^a^ | No^b^ | Yes^a^ | Yes^a^ | 6 |
| Stuart et al. [90], 2012 | Yes^a^ | ? ^c^ | Yes^a^ | ? ^c^ | Yes^a^ | ? ^c^ | ? ^c^ | ? ^c^ | ? ^c^ | ? ^c^ | ? ^c^ | Yes^a^ | Yes^a^ | 5 |
| van Wier et al. [91], 2009 | Yes^a^ | ? ^c^ | No^b^ | ? ^c^ | ? ^c^ | Yes^a^ | No^b^ | Yes^a^ | No^b^ | ? ^c^ | ? ^c^ | Yes^a^ | Yes^a^ | 5 |
| Watson et al. [82], 2015 | Yes^a^ | No^b^ | No^b^ | No^b^ | Yes^a^ | No^b^ | Yes^a^ | Yes^a^ | Yes^a^ | Yes^a^ | ? ^c^ | Yes^a^ | Yes^a^ | 8 |

^a^Criterion was fulfilled.

^b^Criterion was not fulfilled.

^c^Criterion was unclear.
