# Supplementary material for: The Effectiveness of Physical Activity-Promoting Web- and Mobile-Based Distance Weight Loss Interventions on Body Composition in Rehabilitation Settings: Systematic Review, Meta-analysis, and Meta-Regression Analysis
Source: J Med Internet Res. 2022 Mar 24;24(3):e25906. doi: 10.2196/25906 (PMC8990343; doi:10.2196/25906)
Supplement: Multimedia Appendix 4 [file jmir_v24i3e25906_app4.doc]

Multimedia Appendix 4. Results of the meta-regression analysis of BMI, waist circumference, and body fat percentage.

|  | BMI | | | Waist circumference | | | Body fat percent | | |
| --- | --- | --- | --- | --- | --- | --- | --- | --- | --- |
|  |  | | |  | | |  | | |
| Covariate | AICc | Estimate(s) [95% CI] | *P*-value | AICc | Estimate(s) [95% CI] | *P*-value | AICc | Estimate(s) [95% CI] | *P*-value |
| Personal feedback | 60.1 | 0.32 [0.02, 0.62] | .04 | 149.3 | 0.52 [-0.48, 1.52] | .31 | 44.4 | -0.21 [-0.80, 0.39] | .50 |
| - | 61.1 | - | - | 147.7 |  |  | 41.2 |  |  |
| Quality | 61.8 | 0.12 [-0.04, 0.28] | .14 | 149.7 | 0.13 [-0.22, 0.48] | .46 | 41.4 | 0.12 [-0.01, 0.25] | .06 |
| Self–reporting | 62.3 | 0.28 [-0.16, 0.73] | .20 | 148.4 | 0.84 [-0.34, 2.02] | .16 | 42.9 | 0.46 [-0.19, 1.10] | .16 |
| Theory | 63.6 | -0.13 [-0.76, 0.50] | .68 | 150.1 | 0.23 [-1.17, 1.63] | .75 | 44.7 | 0.11 [-0.46, 0.69] | .70 |
| Duration | 63.7 | -0.002 [-0.014, 0.011] | .80 | 150.2 | -0.003 [-0.029, 0.023] | .83 | 44.3 | -0.017 [-0.061, 0.028] | .46 |
| Age | 63.8 | 0.002 [-0.038, 0.043] | .91 | 150.0 | 0.018 [-0.057, 0.093] | .46 | 44.8 | 0.004 [-0.029, 0.037] | .82 |
| Proportion of men | 63.8 | -0.003 [-1.03, 1.03] | .996 | 150.2 | -0.07 [-2.18, 2.06] | .95 | 42.3 | 0.74 [-0.17, 1.67] | .11 |
| Personal goals | 63.8 | -0.004 [-0.42, 0.42] | .99 | 149.8 | -0.40 [-1.51, 0.72] | .48 | 44.7 | -0.12 [-0.69, 0.45] | .68 |
| Prevention | 66.0 | S_a_: -0.13 [-0.95, 0.69]  T_b_: -0.42 [-1.38, 0.53] | .68 | 152.8 | S: -0.04 [-1.73, 1.66]  T: 0.31 [-1.36, 1.99] | .93 | 46.0 | S: 1.29 [-0.12, 2.71]  T: 0.08 [-0.71, 0.88] | .20 |
| Control group | 74.1 | 1_c_: -0.41 [-1.31, 0.49]  2_d_: 0.28 [-0.55, 1.11]  3_e_: -0.28 [-1.39, 0.83]  4_f_: -0.61 [-1.46, 0.24]  5_g_: -0.41 [-1.45, 0.62] | .54 | 158.4 | 1: -0.14 [-2.13, 1.85]  2: 1.14 [-0.79, 3.09]  3: -0.71 [-2.55, 1.12]  4: -0.60 [-2.25, 1.05]  5: -1.21 [-3.68, 1.26] | .54 | 68.8 | 1: 0.07 [-3.08, 3.21]  2: 0.67 [-0.81, 2.14]  3: 0.07 [-0.64, 0.77]  4: 0.09 [-1.14, 1.31]  5: -0.05 [-0.71, 0.61] | .97 |

Prevention compared to primary prevention: _a)_ secondary prevention, _b)_ tertiary prevention;

Control group compared to wait-list or no intervention (0): _c)_ usual care or content of the control group has not been mentioned, _d)_ usual care plus minimal guidance, _e)_ paper instructions, _f)_ paper instructions plus minimal instructions, _g)_ other intervention concerning weight loss
