# Supplementary material for: The Effectiveness of Physical Activity-Promoting Web- and Mobile-Based Distance Weight Loss Interventions on Body Composition in Rehabilitation Settings: Systematic Review, Meta-analysis, and Meta-Regression Analysis
Source: J Med Internet Res. 2022 Mar 24;24(3):e25906. doi: 10.2196/25906 (PMC8990343; doi:10.2196/25906)
Supplement: Multimedia Appendix 5 [file jmir_v24i3e25906_app5.doc]

Multimedia Appendix 5. Main results of the studies included in the meta-analysis of body mass index

| **Primary prevention** | | | | | | | | | | |
| --- | --- | --- | --- | --- | --- | --- | --- | --- | --- | --- |
| **Study** | **Intervention Mean (SD)** | | | **Control Mean (SD)** | | | | | **The difference between groups (95% CI)** | |
|  | Baseline | The end of the intervention | Mean change | Baseline | The end of the intervention | | | Mean change |  | *P*-value |
|  |  |  |  |  |  | | |  |  |  |
| **Chambliss et al. [45] (2010) Basic** | 0 weeks | 12 weeks |  | 0 weeks | 12 weeks | | |  |  |  |
| BMI (kg/m^2^) | 30.1 (2.7) | − | −1.4 (1.2) | 30.7 (2.8) | − | | | 0.0 (0.8) | − | *P < .05* |
| **Chambliss et al. [45] (2010) Enhanced** | 0 weeks | 12 weeks |  | 0 weeks | 12 weeks | | |  |  |  |
| BMI (kg/m^2^) | 30.7 (2.6) | − | −1.3 (1.2) | See control group above | | | | | − | *P < .05* |
| **Collins et al. [47](2012) (Basic)** | 0 weeks | 12 weeks |  | 0 weeks | 12 weeks | | |  |  |  |
| BMI (kg/m^2^) | 32.3 (3.6) | − | −0.7 (1.1) | 32.2 (3.9) | − | | | 0.2 (0.8) | 0.9 (0.5 to 1.2) | *P* < .001 |
| **Collins et al. [47] (2012) (Enhanced)** | 0 weeks | 12 weeks |  | 0 weeks | 12 weeks | | |  |  |  |
| BMI (kg/m^2^) | 32.3 (4.3) | − | −1.0 (1.4) | See control group above | | | | | 3.7 (0.8 to 1.5) | *P* < .001 |
| **Hansen et al. [49] (2012)** | 0 months | 3 months |  | 0 months | 3 months | | |  |  |  |
| BMI (kg/m^2^) | 25.4 (3.8) | 25.3 (SE 0.2) | − | 25.0 (3.8) | 25.0 (SE 0.2) | | | − | − | *P* = .12 |
| **Huber et al. [50] (2015)** | 0 weeks | 12 weeks |  | 0 weeks | 12 weeks | | |  |  |  |
| BMI (kg/m^2^) | 36.5 (4.2) | − | −0.9 (1.2) | 36.1 (3.9) | − | | | −0.3 (0.7) | −0.5 (−1.0 to 0.1) | *P* = .02 |
| **Hunter et al. [51] (2008)** | 0 months | 6 months |  | 0 months | 6 months | | |  |  |  |
| BMI (kg/m^2^) | 29.4 (3.0) | 28.8 (3.3) | −0.5 (1.4) | 29.3 (3.0) | 29.4 (3.0) | | | 0.2 (1.1) | − | *P* < .001 |
| **Lin et al. [52] (2014)** | 0 months | 6 months |  | 0 months | 6 months | | |  |  |  |
| BMI (kg/m^2^) | 28.2 (SE 0.5) | − | −0.6  (SE 0.1) | 28.4 (SE 0.5) | − | | | 0.1 (SE 0.1) | 0.7 (0.4 to 1.0) | *P* < .0001 |
| **Mehring et al. [53] (2013)** | 0 weeks | 12 weeks |  | 0 weeks | 12 weeks | | |  |  |  |
| BMI (kg/m^2^) | 33.6 (7.0) | 32.1 (6.7) | −1.5 (1.4) | 33.3 (5.3) | 32.7 (5.5)  (14.1) | | | −0.6 (1.4) | 0.9 (0.4 to 1.3) | *P* < .001 |
| **Melchart et al. [54] (2017)** | 0 months | 12 months |  | 0 months | 12 months | | |  |  |  |
| BMI (kg/m^2^) | 31.8 (2.0) | − | −3.5 (2.2) | 31.5 (2.0) | − | | | −0.9 (1.4) | − | *P* <.001 |
| **Morgan et al. [55] (2012) SHED-IT Resource** | 0 months | 3 months |  | 0 months | 3 months | | |  |  |  |
| BMI (kg/m^2^) | 32.4 (3.3) | − | −1.0 (95% CI −1.3 to −0.6) | 33.1 (3.9) | − | | | −0.1 (95% CI −0.3 to 0.1) | 0.8 (0.4 to 1.2) | *P* < .0001 |
| **Morgan et al. [55] (2012) SHED-IT Online** | 0 months | 3 months |  | 0 months | 3 months | | |  |  |  |
| BMI (kg/m^2^) | 32.8 (3.4) | − | −1.4 (95% CI −1.7 to −1.0) | See control group above | | | | | 1.2 (0.8 to 1.7) | *P* < .001 |
| **Rogers et al. [57] (2015)** | 0 months | 6 months |  | 0 months | 6 months | | |  |  |  |
| BMI (kg/m^2^) (EN-TECH) | 39.3 (SE 0.8) | 37.3 (SE 1.1) |  | 39.5 (SE 0.7) | 37.2 (SE 1.0) | | |  | − | − |
| **Rogers et al. [57] (2015)** | 0 months | 6 months |  | 0 months | 6 months | | |  |  |  |
| BMI (kg/m^2^) (TECH) | 39.7 (SE 0.8) | 37.8 (SE 1.1) |  | See control group above | | | | | − | − |
| **Sakane et al. [58] (2013)** | 0 weeks | 12 weeks |  | 0 weeks | | 12 weeks |  | |  |  |
| BMI (kg/m^2^) (Web) | 25.6 (3.4) | − | −0.6 (0.7) | 25.4 (2.7) | | − | −0.2 (0.8) | | −0.4 (−0.6 to −0.1) | *P* < .001 |
| **Sakane et al. [58] (2013)** | 0 weeks | 12 weeks |  | 0 weeks | | 12 weeks |  | |  |  |
| BMI (kg/m^2^) (Web + VFA) | 26.2 (3.2) | − | −0.9 (1.1) | See control group above | | | | | −0.8 (−1.1, −0.4) | *P* < .001 |
| **Shuger et al. [59] (2011)** | 0 months | 9 months |  | 0 months | 9 months | | |  |  |  |
| BMI (kg/m^2^) (GWL) | 34.5 (SE 0.9) | 33.8 (SE 0.9) | − | 34.5 (SE 0.9) | 34.2 (SE 0.9) | | | − | − | − |
| **Shuger et al. [59] (2011)** | 0 months | 9 months |  | 0 months | 9 months | | |  |  |  |
| BMI (kg/m^2^) (SWA) | 34.7 (SE 0.9) | 33.6 (SE 0.9) | − | See control group above | | | | | − | − |
| **Shuger et al. [59] (2011)** | 0 months | 9 months |  | 0 months | 9 months | | |  |  |  |
| BMI (kg/m^2^) (GWL+SWA) | 34.4 (SE 0.9) | 32.1 (SE 0.9) | − | See control group above | | | | | − | − |
| **Stephens et al. [60] (2017)** | 0 months | 3 months |  | 0 months | 3 months | | |  |  |  |
| BMI (kg/m^2^) | 29.8 (range 25.5-40.2) | 28.4 (range 24.7-41.3) | − | 27.9 (range 25.0-39.7) | 27.6 (range 25.1-37.7) | | | − | − | *P* = .024 |
| **Secondary and tertiary prevention** | | | | | | | | | | |
| **Aguiar et al. [43] (2016)** | 0 months | 6 months |  | 0 months | 6 months | | |  |  |  |
| BMI (kg/m^2^) | 32.2 (3.5) | ‒ | ‒1.6 (95% CI ‒2.0 to ‒1.2) ^3^ | 32.6 (3.3) | ‒ | | | 0.2 (95% CI ‒0.3 to 0.6) | ‒1.8 (‒2.4 to ‒1.2) | *P* < .001 |
| **Anderson et al. [44] (2010)** | 0 months | 12 months |  | 0 months | 12 months | | |  |  |  |
| BMI (kg/m^2^) | 35.4 (8.6) | 34.5 | ‒ | 33.7 (6.6) | 34.7 | | | ‒ | 0.19 (‒0.5 to 0.8) | *P = .56* |
| **Cho et al. [46] (2018)** | 0 months | 4 weeks |  | 0 months | 4 weeks | | |  |  |  |
| BMI (kg/m^2^) | 28.4 (3.6) | 27.7 (3.5) | ‒ | 27.3 (2.4) | 27.1 (2.4) | | | ‒ | ‒ | ‒ |
| **Hageman et al. [48] (2014)** | 0 months | 12 months |  | 0 months | 12 months | | |  |  |  |
| BMI (kg/m^2^) (web-based) | 28.6 (5.1) | − | −0.8 (1.3) | 29.7 (4.7) | − | | | −0.3 (1.6) | −0.4 (−1.0 to 0.1) | *P* = .047 |
| **Hageman et al. [48] (2014)** | 0 months | 12 months |  | 0 months | 12 months | | |  |  |  |
| BMI (kg/m^2^) (print-mailed) | 30.5 (5.4) | − | −0.6 (1.6) | See control group above | | | | | −0.1 (−0.6 to 0.4) | *P* = .056 |
| **Rimmer et al. [56] (2013) POWERS** | 0 months | 9 months |  | 0 months | | 9 months |  | |  |  |
| BMI (kg/m^2^) | 33.4 | 32.7 | − | 32.0 | | 33.0 |  | | − | *P* < .01 |
| **Rimmer et al. [56] (2013) POWERS(plus)** | 0 months | 9 months |  | 0 months | | 9 months |  | |  |  |
| BMI (kg/m^2^) | 31.4 | 31.2 | − | See control group above | | | | | − | *P* = .04 |
| **Watson et al. [61] (2015)** | 0 months | 12 months |  | 0 months | | 12 months |  | |  |  |
| BMI (kg/m^2^) | 32.9 (3.1) | − | –0.78 (95% CI–1.26, –0.31) | 32.4 (2.7) | | − | –0.65 (95% CI –1.12, −0.19)_a_ | | –0.10 (–0.75, 0.55) | *P* = .76 |

SD = Standard deviation; SE = Standard error

**_a)_** In the study of Watson et al. there has been an obvious character error in the upper limit of the 95% CI. The character error has been corrected.
