# Supplementary material for: The Effectiveness of Physical Activity-Promoting Web- and Mobile-Based Distance Weight Loss Interventions on Body Composition in Rehabilitation Settings: Systematic Review, Meta-analysis, and Meta-Regression Analysis
Source: J Med Internet Res. 2022 Mar 24;24(3):e25906. doi: 10.2196/25906 (PMC8990343; doi:10.2196/25906)
Supplement: Multimedia Appendix 6 [file jmir_v24i3e25906_app6.doc]

Multimedia Appendix 6. Main results of the studies included in the meta-analysis of waist circumference

| **Primary prevention** | | | | | | | | | | |
| --- | --- | --- | --- | --- | --- | --- | --- | --- | --- | --- |
| **Study** | **Intervention Mean (SD)** | | | **Control Mean (SD)** | | | | | **The difference between groups (95% CI)** | |
|  | Baseline | The end of the intervention | Mean change | Baseline | The end of the intervention | | | Mean change |  | *P*-value |
|  |  |  |  |  |  | | |  |  |  |
| **Chambliss et al. [45] (2010) Basic** | 0 weeks | 12 weeks |  | 0 weeks | 12 weeks | | |  |  |  |
| Waist circumference (cm) | 97.1 (9.8) | − | −3.4 (4.6) | 100.1 (11.3) | − | | | −0.6 (5.2) | − | *P* = .04 |
| **Chambliss et al. [45] (2010) Enhanced** | 0 weeks | 12 weeks |  | 0 weeks | 12 weeks | | |  |  |  |
| Waist circumference (cm) | 98.2 (9.4) | − | −2.8 (5.4) | See control group above | | | | | − | − |
| **Collins et al. [47] (2012) Basic** | 0 weeks | 12 weeks |  | 0 weeks | 12 weeks | | |  |  |  |
| Waist circumference (cm) | 106.9 (9.8) | − | −2.6 (4.0) | 107.2 (10.4) | − | | | 0.3 (3.1) | 2.4 (1.5 to 4.3) | *P* < .001 |
| **Collins et al. [47] (2012) Enhanced** | 0 weeks | 12 weeks |  | 0 weeks | 12 weeks | | |  |  |  |
| Waist circumference (cm) | 106.6 (12.5) | − | −3.2 (5.0) | See control group above | | | | | 3.7 (2.1 to 4.8) | *P* < .001 |
| **Haapala et al. [64] (2009)** | 0 months | 12 months |  | 0 months | 12 months | | |  |  |  |
| Waist circumference (cm) | 98.5 (10.3) | 91.3 (11.7) | −6.3 (5.3) | 96.6 (10.4) | 93.3 (11.1) | | | −2.4 (5.4) | − | *P* < .001 |
| **Hansen et al. [49] (2012)** | 0 months | 3 months |  | 0 months | 3 months | | |  |  |  |
| Waist circumference (cm) | 90.1 (12.0) | 90.0 (0.5) | − | 89.6 (11.8) | 89.1 (0.5) | | | − | − | *P* = .34 |
| **Hunter et al. [51] (2008)** | 0 months | 6 months |  | 0 months | 6 months | | |  |  |  |
| Waist circumference (cm) | 94.5 (11.0) | 92.2 (11.6) | −2.1 (4.3) | 94.2 (10.9) | 93.4 (12.8) | | | −0.4 (3.8) | − | *P* < .001 |
| **Huber et al. [50] (2015)** | 0 weeks | 12 weeks |  | 0 weeks | 12 weeks | | |  |  |  |
| Waist circumference (cm) | 108.6 (9.3) | − | −3.2 (3.6) | 112.4 (13.3) | − | | | −1.9 (2.6) | −1.3 (−2.7 to 0.1) | *P* = .072 |
| **Lin et al. [52] (2014)** | 0 months | 6 months |  | 0 months | 6 months | | |  |  |  |
| Waist circumference (cm) | 94.1 (1.32) | − | −2.7  (0.4) | 95.3 (1.35) | − | | | -0.1 (0.4) | 2.6 (1.4 to 3.8) | *P* < .0001 |
| **Mehring et al. [53] (2013)** | 0 weeks | 12 weeks |  | 0 weeks | 12 weeks | | |  |  |  |
| Waist circumference (cm) | 110.9 (17.6) | 104.4 (15.5) | -6.9 (6.9) | 107.3 (13.7) | 106.0  (14.1) | | | −2.4 (5.0) | 4.5 (2.3 to 6.7) | *P* < .001 |
| **Melchart et al. [54] (2017)** | 0 months | 12 months |  | 0 months | 12 months | | |  |  |  |
| Waist circumference (cm) | 103.7 (8.8) | − | −11.8 (7.6) | 103.2 (9.9) | − | | | −5.3 (5.3) | − | *P* < .001 |
| **Morgan et al. [55] (2012) SHED-IT Resource** | 0 months | 3 months |  | 0 months | 3 months | | |  |  |  |
| Waist circumference (cm) | 112.1 (9.3) | − | −2.6 (3.2) | 113.4 (9.9) | − | | | −0.4 (2.4) | 2.2 (0.9 to 3.4) | *P* < .0001 |
| **Morgan et al. [55] (2012) SHED-IT Online** | 0 months | 3 months |  | 0 months | 3 months | | |  |  |  |
| Waist circumference (cm) | 113.0 (10.2) | − | −4.1 (4.1) | See control group above | | | | | 3.7 (2.4 to 5.0) | *P* < .0001 |
| **Rogers et al. [57] (2015) (EN-TECH)** | 0 months | 6 months |  | 0 months | 6 months | | |  |  |  |
| Waist circumference (cm) | 122.3 (SE 2.5) | 115.3 (SE 3.3) |  | 119.1 (SE 2.4) | 115.1 (SE 3.0) | | |  | − | *P* = .0002 |
| **Rogers et al. [57] (2015)** **(TECH)** | 0 months | 6 months |  | 0 months | 6 months | | |  |  |  |
| Waist circumference (cm) | 122.8 (SE 2.6) | 118.2 (SE 3.2) |  | See control group above | | | | | − | *P* = .0002 |
| **Sakane et al. [58] (2013)** **(Web)** | 0 weeks | 12 weeks |  | 0 weeks | | 12 weeks |  | |  |  |
| Waist circumference (cm) | 89.4 (8.5) | − | −1.6 (2.6) | 88.1 (7.6) | | − | 0.1 (3.6) | | −1.6 (−2.8 to −0.5) | *P* < .001 |
| **Sakane et al. [58] (2013)** **(Web + VFA)** | 0 weeks | 12 weeks |  | 0 weeks | | 12 weeks |  | |  |  |
| Waist circumference (cm) | 91.5 (8.6) | − | −3.2 (3.3) | See control group above | | | | | −3.3 (−4.5, −2.1) | *P* < .001 |
| **Shuger et al. [58] (2011)** **(GWL)** | 0 months | 9 months |  | 0 months | 9 months | | |  |  |  |
| Waist circumference (cm) | 108.3 (SE 2.2) | 105.9 (SE 2.2) | − | 106.3 (SE 2.2) | 102.8 (SE 2.3) | | | − | − | − |
| **Shuger et al. [59] (2011)** **(SWA)** | 0 months | 9 months |  | 0 months | 9 months | | |  |  |  |
| Waist circumference (cm) | 105.9 (SE 2.2) | 102.3 (SE 2.2) | − | See control group above | | | | | − | − |
| **Shuger et al. [59] (2011)** **(GWL+SWA)** | 0 months | 9 months |  | 0 months | 9 months | | |  |  |  |
| Waist circumference (cm) | 106.0 (SE 2.2) | 99.3 (SE 2.2) | − | See control group above | | | | | − | − |
| **Stephens et al. [60] (2017)** | 0 months | 3 months |  | 0 months | 3 months | | |  |  |  |
| Waist circumference (cm) | 95.8 (range 82.5-120) | 92.3 (range 77-122) | − | 93.3 (range 81-120) | 92.3 (range 81-117) | | | − | − | *P* < .01 |
| **van Wier et al. [70] (2009) Phone** | 0 months | 6 months |  | 0 months | 6 months | | |  |  |  |
| Waist circumference (cm) | 102.6 (10.0) | 98.6 (10.3) | − | 101.5 (9.8) | 99.5 (10.0) | | | − | −1.9 (−2.7 to −1.0) | *P* < .001 |
| **van Wier et al. [70] (2009) Internet** | 0 months | 6 months |  | 0 months | 6 months | | |  |  |  |
| Waist circumference (cm) | 101.5 (10.3) | 98.2 (10.2) | − | See control group above | | | | | −1.2 (−2.1 to −0.4) | *P* < .01 |
| **Secondary and tertiary prevention** | | | | | | | | | | |
| **Aguiar et al. [43] (2016)** | 0 months | 6 months |  | 0 months | 6 months | | |  |  |  |
| Waist circumference (cm) | 112.0 (9.0) | ‒ | ‒4.9 (95% CI ‒6.3 to 3.5) | 112.9 (8.5) | ‒ | | | 0.44 (95% CI ‒1.0 to 1.9) | ‒5.35 (‒7.35 to ‒3.36) | *P* < .001 |
| **Chen et al. [62] (2012)** | 0 months | 3 months |  | 0 months | 3 months | | |  |  |  |
| Waist circumference (cm) | 91.9 (11.0) | 88.4 (11.2) | −3.5 | 88.9 (10.0) | 88.3 (10.4) | | | −3.1 | − | *P* = .05 |
| **Eakin et al. [63] (2014)** | 0 months | 18 months |  | 0 months | 18 months | | |  |  |  |
| Waist circumference (cm) | 109.3 (13.3) | 107.7 (13.9) | −2.2 (6.0) | 110.0 (14.0) | 109.6 (14.4) | | | −0.4 (5.8) | −1.8  (−3.2 to −0.3) ^1^ | *P* = .016 |
| **Hageman et al. [48] (2014)** **(web-based)** | 0 months | 12 months |  | 0 months | 12 months | | |  |  |  |
| Waist circumference (cm) | 95.2 (11.9) | 91.9 (12.1) | −3.2 (4.4) | 97.5 (11.4) | 96.0 (11.1) | | | −1.5 (5.0) | −1.6 (−3.1 to −0.1) | *P* = .017 |
| **Hageman et al. [48] (2014)** **(print-mailed)** | 0 months | 12 months |  | 0 months | 12 months | | |  |  |  |
| Waist circumference (cm) | 99.3 (13.0) | 95.7 (12.7) | −3.6 (5.4) | See control group above | | | | | −1.6 (−3.1 to −0.1) | *P* = .016 |
| **Harrigan et al. [65] (2016) In-person** | 0 months | 6 months |  | 0 months | 6 months | | |  |  |  |
| Waist circumference (cm) | 101.3 (12.8) | 92.5 (13.3) | −7.5 (6.2) | 99.4 (15.4) | 96.0 (15.3) | | | −2.6 (5.9) | − | *P* = .002 |
| **Harrigan et al. [65] (2016) Telephone** | 0 months | 6 months |  | 0 months | 6 months | | |  |  |  |
| Waist circumference (cm) | 98.3 (10.7) | 91.1 (11.1) | −7.2 (6.9) | See control group above | | | | | − | *P* = .005 |
| **Karhula et al. [66] (2015) Heart disease** | 0 months | 12 months |  | 0 months | 12 months | | |  |  |  |
| Waist circumference (cm) | 101.5 (12.7) | 100.6 (12.1) | −0.9 (4.6) | 97.6 (1.4) | 98.7 (1.8) | | | 1.1 (11.1) | −1.5 (−3.6 to 2.2) | *P*  = .15 |
| **Karhula et al. [66] (2015) Diabetes** | 0 months | 12 months |  | 0 months | 12 months | | |  |  |  |
| Waist circumference (cm) | 107.8 (1.1) | 105.8 (1.0) | −2.0 (4.4) | 107.4 (2.2) | 107.1 (2.2) | | | −0.3 (4.5) | −1.7 (−3.0 to −0.4) | *P*  < .01 |
| **Ligibel et al. [67]**  **(2012)** | 0 weeks | 16 weeks |  | 0 weeks | 16 weeks | | |  |  |  |
| Waist circumference (cm) | 96.7 (20.0) | - | 1.4 (13.2) | 94.0 (16.1) | - | | | 2.3 (9.4) | − | *P* = .70 |
| **Reeves et al. [68] (2017)** | 0 months | 6 months |  | 0 months | 6 months | | |  |  |  |
| Waist circumference (cm) | 101.5 (10.8) | ‒ | ‒3.6 (95% CI ‒5.2 to ‒2.0) | 103.0 (10.2) | ‒ | | | 0.6 (95% CI ‒1.0 to 2.3) | ‒4.0 (95% CI ‒6.6 to ‒1.3) | *P* = .004 |
| **Stuart et al. [69] (2012)** | 0 weeks | 12 weeks |  | 0 weeks | 12 weeks | | |  |  |  |
| Waist circumference (cm) | 104. 9  (SE 1.30) | 101.9  (SE 1.32) | − | 106.4  (SE 1.35) | 104.2  (SE 1.36) | | | − | − | *P* = .58 |
| **Watson et al. [61] (2015)** | 0 months | 12 months |  | 0 months | 12 months | | |  |  |  |
| Waist circumference (cm) | 103.5 (11.2) | − | –2.31 (95% CI  –3.84, –0.79) | 102.5 (9.5) | − | | | –1.80 (95% CI  –3.02, –0.58) | –0.42 (–2.29, 1.45) | *P* = .66 |

SD = Standard deviation; SE = Standard error
