# Supplementary material for: The Effectiveness of Physical Activity-Promoting Web- and Mobile-Based Distance Weight Loss Interventions on Body Composition in Rehabilitation Settings: Systematic Review, Meta-analysis, and Meta-Regression Analysis
Source: J Med Internet Res. 2022 Mar 24;24(3):e25906. doi: 10.2196/25906 (PMC8990343; doi:10.2196/25906)
Supplement: Multimedia Appendix 7 [file jmir_v24i3e25906_app7.doc]

Multimedia Appendix 7. Main results of the studies included in the meta-analysis of body fat percent

| **Primary prevention** | | | | | | | | |
| --- | --- | --- | --- | --- | --- | --- | --- | --- |
| **Study** | **Intervention Mean (SD)** | | | **Control Mean (SD)** | | | **The difference between groups** | |
|  | Baseline | The end of the intervention | Mean change | Baseline | The end of the intervention | Mean change |  | *P*-value |
|  |  |  |  |  |  |  |  |  |
| **Hansen et al. [49] (2012)** | 0 months | 3 months |  | 0 months | 3 months |  |  |  |
| Body fat (%) | 30.4 (8.2) | 30.4 (SE 0.3) | − | 30.5 (8.0) | 30.5 (SE 0.3) | − | − | *P* = .87 |
| **Hunter et al. [51] (2008)** | 0 months | 6 months |  | 0 months | 6 months |  |  |  |
| Body fat percentage (%) | 34.5 (6.8) | 33.9 (7.3) | −0.4 (3.1) | 34.2 (6.9) | 34.7 (7.0) | 0.6 (2.0) | − | *P* < .001 |
| **Lin et al. [52] (2014)** | 0 months | 6 months |  | 0 months | 6 months |  |  |  |
| Fat percent (%) | 34.3  (SE 0.9) | − | −0.7 (SE 0.19) | 34.8  (SE 0.9) | − | 0.4 (SE 0.2) | 1.0 (0.5 to 1.6) | *P* = .0003 |
| **Morgan et al. [55] (2012) (SHED-IT Resource** | 0 months | 3 months |  | 0 months | 3 months |  |  |  |
| Fat mass (%) | 32.7 (5.6) | − | −2.3 (2.8) | 32.1 (5.4) | − | −0.9 (1.6) | 1.4 (0.5 to 2.4) | *P* = .0015 |
| **Morgan et al. [55] (2012) SHED-IT Online** | 0 months | 3 months |  | 0 months | 3 months |  |  |  |
| Fat mass (%) | 31.7 (4.5) | − | −2.5 (2.8) | See control group above | | | 1.6 (0.7 to 2.6) | *P* = .0015 |
| **Rogers et al. [57] (2015)** | 0 months | 6 months |  | 0 months | 6 months |  |  |  |
| Percent body fat (%) (EN-TECH) | 45.4 (SE 1.1) | 42.9 (SE 1.6) | − | 46.2 (SE 1.1) | 44.2 (SE 1.5) | − | − | − |
| **Rogers et al. [57] (2015)** | 0 months | 6 months |  | 0 months | 6 months |  |  |  |
| Percent body fat (%) (TECH) | 45.8 (SE 1.2) | 44.4 (SE 1.6) | − | See control group above | | | − | − |
| **Shuger et al. [59] (2011)** | 0 months | 9 months |  | 0 months | 9 months |  |  |  |
| % body fat (GWL) | 36.7 (SE 0.8) | 32.4 (SE 0.9) | − | 36.4 (SE 0.8) | 33.1 (SE 0.9) | − | − | − |
| **Shuger et al. [59] (2011)** | 0 months | 9 months |  | 0 months | 9 months |  |  |  |
| % body fat (SWA) | 36.6 (SE 0.8) | 32.4 (SE 0.9) | − | See control group above | | | − | − |
| **Shuger et al. [59] (2011)** | 0 months | 9 months |  | 0 months | 9 months |  |  |  |
| % body fat (GWL+SWA) | 36.5 (SE 0.8) | 31.4 (SE 0.8) | − | See control group above | | | − | − |
| **Secondary and tertiary prevention** | | | | | | | | |
| **Aguiar et al. [43] (2016)** | 0 months | 6 months |  | 0 months | 6 months |  |  |  |
| Body fat percentage (%) | 30.6 (5.6) | ‒ | ‒2.1 (95% CI ‒3.1 to ‒1.1) | 33.0 (5.7) | ‒ | 0.2 (95% CI ‒0.8 to 1.2) | ‒2.3 3.7 to ‒0.9) | *P* = .002 |
| **Devi et al. [71] (2014)** | 0 weeks | 6 weeks |  | 0 weeks | 6 weeks |  |  |  |
| Body fat (%) | 38.8 (10.8) | 38.4 (11.5) | −0.4 (7.7) | 36.3 (8.0) | 37.0 (7.1) | 0.7 (6.4) | −1.1 (−1.8 to −0.2) | *P* = 0.49 |
| **Harrigan et al. [65] (2016) In-person** | 0 months | 6 months |  | 0 months | 6 months |  |  |  |
| Fat percent (%) | 43.3 (4.8) | 40.4 (5.5) | −3.2 (−4.4 to −2.1) | 42.7 (6.5) | 40.5 (6.6) | −1.7 (−2.8 to −0.5) | − | *P* = 0.05 |
| **Harrigan et al. [65] (2016) Telephone** | 0 months | 6 months |  | 0 months | 6 months |  |  |  |
| Fat percent (%) | 43.3 (4.6) | 40.8 (5.7) | −2.4 (−3.7 to −1.2) | See control group above | | | − | *P* = 0.37 |
| **Matthews et al. [72] (2006)** | 0 weeks | 12 weeks |  | 0 weeks | 12 weeks |  |  |  |
| Body fat %) | 39.9 (6.8) | 39.7 (6.3) | −0.2 (1.6) | 42.7 (7.3) | 43.1 (6.9) | 0.4 (1.9) | − | *P* = 0.15 |
| **Reeves et al. [68] (2017)** | 0 months | 6 months |  | 0 months | 6 months |  |  |  |
| Fat (% of bodyweight) | 39.3 (5.1) | ‒ | ‒2.6 (95% CI ‒3.6 to ‒1.6) | 39.0 (4.7) | ‒ | ‒0.9 (95% CI ‒1.9 to 0.2) | ‒1.6 (‒3.4 to 0.1) | *P* = .067 |

SD = Standard deviation; SE = Standard error
